# Increased CD8+ T Cell Response to Epstein-Barr Virus Lytic Antigens in the Active Phase of Multiple Sclerosis

**DOI:** 10.1371/journal.ppat.1003220

**Published:** 2013-04-11

**Authors:** Daniela F. Angelini, Barbara Serafini, Eleonora Piras, Martina Severa, Eliana M. Coccia, Barbara Rosicarelli, Serena Ruggieri, Claudio Gasperini, Fabio Buttari, Diego Centonze, Rosella Mechelli, Marco Salvetti, Giovanna Borsellino, Francesca Aloisi, Luca Battistini

**Affiliations:** 1 Neuroimmunology Unit, Fondazione Santa Lucia, (I.R.C.C.S.), Rome, Italy; 2 Department of Cell Biology and Neuroscience, Istituto Superiore di Sanità, Rome, Italy; 3 Department of Infectious, Parasitic and Immune-mediated Diseases, Istituto Superiore di Sanità, Rome, Italy; 4 Department of Neurology and Psychiatry, Sapienza University of Rome, Rome, Italy; 5 Department of Neurosciences, S Camillo Forlanini Hospital, Rome, Italy; 6 Department of Neurosciences, University Tor Vergata, Rome, Italy; 7 Centre for Experimental Neurological Therapies, S. Andrea Hospital, Faculty of Medicine and Psychology, Sapienza University of Rome, Rome, Italy; Emory University, United States of America

## Abstract

It has long been known that multiple sclerosis (MS) is associated with an increased Epstein-Barr virus (EBV) seroprevalence and high immune reactivity to EBV and that infectious mononucleosis increases MS risk. This evidence led to postulate that EBV infection plays a role in MS etiopathogenesis, although the mechanisms are debated. This study was designed to assess the prevalence and magnitude of CD8+ T-cell responses to EBV latent (EBNA-3A, LMP-2A) and lytic (BZLF-1, BMLF-1) antigens in relapsing-remitting MS patients (n = 113) and healthy donors (HD) (n = 43) and to investigate whether the EBV-specific CD8+ T cell response correlates with disease activity, as defined by clinical evaluation and gadolinium-enhanced magnetic resonance imaging. Using HLA class I pentamers, lytic antigen-specific CD8+ T cell responses were detected in fewer untreated inactive MS patients than in active MS patients and HD while the frequency of CD8+ T cells specific for EBV lytic and latent antigens was higher in active and inactive MS patients, respectively. In contrast, the CD8+ T cell response to cytomegalovirus did not differ between HD and MS patients, irrespective of the disease phase. Marked differences in the prevalence of EBV-specific CD8+ T cell responses were observed in patients treated with interferon-β and natalizumab, two licensed drugs for relapsing-remitting MS. Longitudinal studies revealed expansion of CD8+ T cells specific for EBV lytic antigens during active disease in untreated MS patients but not in relapse-free, natalizumab-treated patients. Analysis of post-mortem MS brain samples showed expression of the EBV lytic protein BZLF-1 and interactions between cytotoxic CD8+ T cells and EBV lytically infected plasma cells in inflammatory white matter lesions and meninges. We therefore propose that inability to control EBV infection during inactive MS could set the stage for intracerebral viral reactivation and disease relapse.

## Author Summary

There is general consensus that multiple sclerosis (MS) is associated with Epstein-Barr virus (EBV) infection but the mechanistic links are still debated. EBV is a B-lymphotropic herpesvirus widespread in the human population and normally contained as a persistent, asymptomatic infection by immune surveillance. However, EBV can cause infectious mononucleosis, is associated with numerous human malignancies, and is implicated in some common autoimmune diseases. While EBV infection alone cannot explain MS development, it has been postulated that in susceptible individuals alterations in the mechanisms regulating the immune response to the virus may contribute to MS pathogenesis. Here, we show that MS patients with inactive disease exhibit a lower CD8+ T-cell response to EBV when compared to healthy donors and active MS patients while the latter have a higher frequency of CD8+ T cells specific for EBV lytic antigens. Therapy with interferon-β and natalizumab, two treatments for relapsing-remitting MS, was associated with marked changes in the EBV specific CD8+ T cell response. We also demonstrate that one of the EBV lytic antigens recognized by CD8+ T cells expanding in the blood during active MS is expressed in the inflamed MS brain. Our results support a model of MS pathogenesis in which EBV infection and reactivation in the CNS stimulates an immunopathological response and suggest that antiviral or immunomodulatory therapies aimed at restoring the host-EBV balance could be beneficial to MS patients.

## Introduction

Multiple sclerosis (MS) is the most common chronic inflammatory disease of the central nervous system (CNS) causing demyelination, neurodegeneration and disability. In most cases, MS is characterized by a relapsing-remitting course at onset which eventually develops into a progressive form; more rarely MS manifests as a primary progressive disease [1]. Immunomodulating and immunosuppressive drugs can reduce but not halt the disease process. Both the etiology and pathogenic mechanisms of MS are poorly understood. Genetic and environmental factors have been implicated in MS development, but the identity of the antigens (self or non-self) promoting chronic CNS inflammation remains elusive [2]. Several viruses have been linked to MS; however, Esptein-Barr virus (EBV) shows the strongest association with the disease [3]–[5]. EBV is a B-lymphotropic DNA herpesvirus that infects 95–98% of individuals worldwide, establishes a life-long, generally asymptomatic infection in B cells, and is the cause of infectious mononucleosis and of several lymphatic and non-lymphatic malignancies [6]. EBV has also been implicated in common autoimmune diseases, like systemic lupus erythematosus and rheumatoid arthritis [7], [8]. Numerous studies have consistently demonstrated a higher prevalence of EBV infection and higher titers of antibodies to EBV antigens, in particular to EBV nuclear antigen-1 (EBNA-1), in young and adult MS patients compared to age-matched, healthy individuals [9]–[14]. It has also been shown that high titers of anti-EBNA-1 antibodies prior to MS onset [15] or at the time of a clinically isolated syndrome [16] and a previous history of infectious mononucleosis [17] increase the risk of developing MS. Furthermore, MS patients have higher frequencies of CD4+ T cells specific for EBNA-1 relatively to healthy, seropositive individuals [18], while EBV-specific CD8+ T-cell responses in MS have been reported to be increased or decreased in different studies [19]–[23].

Although enhanced immune reactivity to EBV in MS suggests perturbed EBV infection, it is debated whether and how this can induce or influence the disease. EBV infection could contribute to MS through multiple mechanisms, including molecular mimicry, immortalization of autoantibody-producing B cell clones, and immunopathology [3], [24]. It has been shown that CD4+ T cells from some MS patients cross-react with EBV and myelin antigens but the relevance of this finding to disease pathogenesis is still unclear [25], [26]. EBV DNA load in the peripheral blood does not differ significantly between MS patients and healthy donors (HD) [16], [18], [23] and the possibility that a persistent EBV infection in the CNS drives an immunopathological response that damages myelin and neural cells is reasonable but remains controversial [27]. While several studies report absence of EBV in MS brain lesions [28]–[31], we [32]–[34] and others [35] have shown that an abnormally high proportion of B cells infiltrating the MS brain are latently infected with EBV. We have also shown that ectopic B-cell follicles present in the inflamed meninges of patients with secondary progressive MS harbour EBV infected B cells and that EBV can reactivate in plasma cells in immunologically active white matter lesions and meningeal B-cell follicles [32]–[34].

If MS were the result of an immunopathological response aimed at eliminating a persistent EBV infection in the CNS, a positive correlation should be found between disease activity assessed by magnetic resonance imaging (MRI) or clinical progression and immune response to EBV. In support of this hypothesis, it has been shown that serum levels of EBNA-1 IgG positively correlate with gadolinium-enhancing MRI lesions (characteristic of acute inflammation), lesion size and Expanded Disability Status Scale (EDSS) in patients with MS [36] and in patients with a clinically isolated syndrome who develop definite MS [16]. Another study reported higher disease activity on MRI in a subgroup of relapsing-remitting MS patients with stable levels of IgG specific for EBV early antigens expressed during the lytic cycle [37]. It has also been shown that CD8+ T-cell responses toward pooled EBV latent and lytic antigens in the blood of MS patients are high early in MS course and decrease during disease progression suggesting a possible association with more frequent episodes of CNS inflammation in early disease phases [21]. However, it is not known whether changes in the CD8+ T cell response to individual EBV latent and/or lytic antigens are associated with active and inactive MS phases. To address this issue, we have used pentamer staining to characterize the CD8+ T-cell response to EBV in the peripheral blood of patients with relapsing-remitting MS, both untreated and treated, and HLA-matched controls. Positivity to pentamer staining was then correlated with disease activity and inactivity, as assessed by clinical criteria and MRI of the brain. Our study reveals a lower prevalence of the CD8+ T cell response to EBV in inactive MS patients, a higher frequency of CD8+ T cells specific for EBV lytic and latent antigens during active and inactive disease, respectively, and marked changes in the EBV-specific CD8+ T cell response during treatment with approved disease-modifying drugs, such as interferon-β (IFN-β) and natalizumab. By analyzing post-mortem MS brain tissue, we demonstrate that the same EBV lytic antigen eliciting a higher CD8+ T cell response in the peripheral blood during active MS is expressed in inflammatory white matter lesions and meninges. We also show interactions between CNS-infiltrating cytotoxic CD8+ T cells and EBV lytically infected plasma cells, further supporting the link between EBV reactivation, higher cytotoxic immune responses to EBV lytic antigens and MS exacerbations.

## Results

The EBV-specific CD8+ T-cell response was studied with the pentamer technology in 43 HD and 113 patients (79 untreated and 34 treated) with relapsing-remitting MS who were selected according to their HLA genotype (HLA-A*0201 and/or HLA-B*0801) (Table 1). The choice of pentamers was guided by the high frequency of HLA-A2 family alleles in Caucasians, by previous characterization of the immunodominant peptide epitopes from EBV latent and lytic proteins that are most frequently recognized by CD8+ T cells [38]-[40], and by the possibility to evaluate the CD8+ T cell response to at least one viral latent and one viral lytic protein in the same subject. We therefore studied CD8+ T cell reactivities to the immunodominant peptides of LMP-2A (EBV latent antigen) and BMLF-1 (EBV early lytic antigen) restricted through the HLA-A*0201 allele, and of EBNA-3A (EBV latent antigen) and BZLF-1 (EBV immediate early lytic antigen) restricted through the HLA-B*0801 allele. As control for anti-viral MHC class I restricted CD8+ T cell responses we selected a HLA-A*0201 pentamer coupled with a peptide from cytomegalovirus (CMV) pp65 protein.

**Table 1:** Demographic and clinical characteristics of analyzed subjects.

	Females/males (ratio)	Age (years) Median (range)	Duration of MS (years) Median (range)	EDSS Median (range)	Percentage of HLA-A2+ subjects	Percentage of HLA-B8+ subjects
HD (n = 43)	34/9 (3.8∶1)	40 (25–58)	N/A	N/A	79%	29%
MS (n = 79) untreated	56/23 (2.4∶1)	37 (19–61)	6 (0–27)	2 (0–6)	57%	43%
MS IFNβ (n = 20)	16/4 (4∶1)	40 (30–61)	4 (1–11)	2 (1–4)	75%	25%
MS NTZ^a^ (n = 14)	11/3 (3.6∶1)	38 (28–53)	12 (8–16)^a^	2.5 (0–5.5)	57%	43%

a Months.

### Prevalence and frequency of EBV-specific CD8+ T cells in healthy donors and in untreated MS patients with active or inactive disease

**Figure 1:**
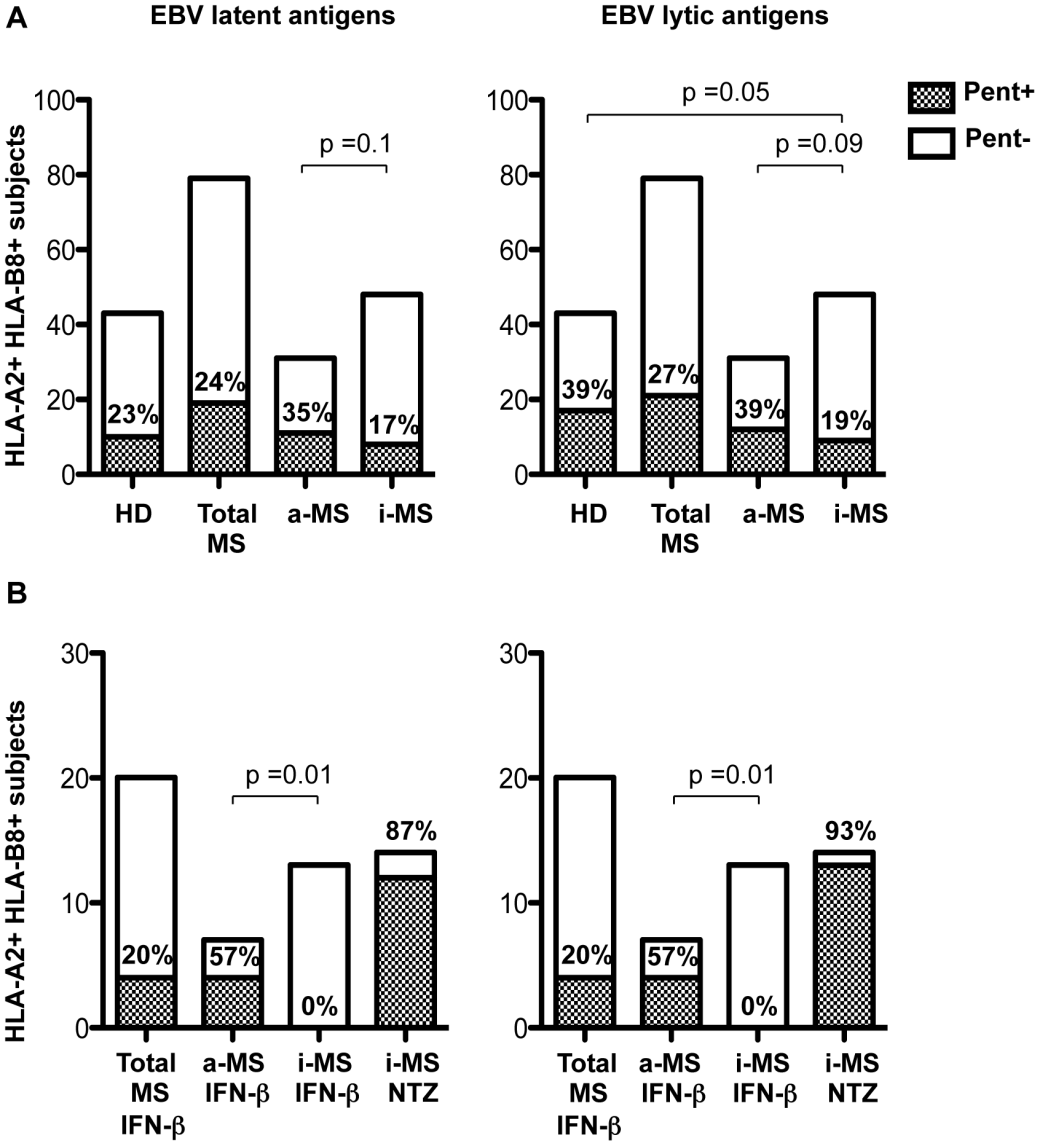
Prevalence of EBV-specific CD8+ T cell responses in HD and MS patients. (A) HLA-A*0201 and HLA-B*0801 pentamer+ CD8+ T cells specific for peptides from EBV latent (left panel) and lytic (left) proteins were investigated in HLA matched HD (n = 43), total untreated MS patients (n = 79) and the same patients subdivided into active (n = 31) and inactive (n = 48) MS patients (a-MS and i-MS, respectively) based on clinical and MRI criteria, as shown in Table 2. (B) IFN-β (n = 20) and natalizumab (NTZ) (n = 14) treated patients were analyzed as whole population or subdivided into active and inactive patients, as above. The numbers within or above the columns correspond to the percentages of individuals with detectable EBV-pentamer+ CD8+ T cells (grey columns) among the total donors tested (white columns); p values were calculated with Pearson's chi-squared test.

Freshly isolated PBMC from HD (n = 43) and untreated MS patients (n = 79) were stained with the above mentioned fluorochrome-labeled HLA-A*0201 and/or HLA-B*0801/viral peptide epitope pentamers. We first examined the prevalence of EBV-specific CD8+ T cell responses in our cohort, namely the proportion of individuals with detectable pentamer+ CD8+ T cells (Fig. S1). Positive pentamer staining specific for at least one EBV epitope was found in a similar proportion of HD (39%) and untreated MS patients (33%). The prevalence of CD8+ T cell responses to EBV latent and lytic antigens was similar in HD and untreated MS patients (Figure 1 A). In HLA-A2+ subjects where both EBV- and CMV-specific CD8+ T cell responses could be evaluated, no differences in the response to either virus were found between HD (n = 34) and untreated MS patients (n = 45) (Figure S2A).

We then explored whether the prevalence of EBV-specific CD8+ T-cell responses could be related to disease activity. Untreated MS patients were subdivided in two groups consisting of 31 active and 48 inactive patients, as defined by presence and absence of clinical relapses and acute brain inflammation assessed with gadolinium-enhanced MRI, respectively (Table 2). There was a similar prevalence of latent and lytic antigen-specific CD8+ T cell responses in HD and active MS patients but a significantly lower prevalence of lytic antigen-specific CD8+ T cell responses in inactive MS patients than HD (19% versus 39%, p = 0.05). Inactive MS patients also tended to have a lower prevalence of latent and lytic antigen-specific CD8+ T cell responses when compared with active MS patients (17% versus 35%, p = 0.1 and 19% versus 39%, p = 0.09, respectively) (Figure 1A). In contrast, no differences were found among HD, inactive MS and active MS patients in the CD8+ T cell response to CMV (Figure S2B). These findings therefore indicated a weaker immune response against EBV, particularly against lytic cycle antigens, in inactive MS patients compared to HD and active MS patients. Of note, 39% of active MS patients, but only 15% of inactive MS patients with a detectable EBV-specific CD8+ T cell response had a disease duration longer than 8 years, suggesting a decay with time of the EBV-specific immune response associated with inactive disease.

**Table 2:** Characterization of active and inactive MS patients.

	Patients studied with MRI	Patients with active MRI lesions and clinical relapses	Patients with active MRI lesions and without clinical relapses	Patients without active MRI lesions and with clinical relapses	Patients without active MRI lesions and clinical relapses	Patients without clinical relapses not studied with MRI
Active MS (n = 31)	29/31	24/31	2/31	3/31	-	-
Inactive MS (n = 48)	31/48	-	-	-	31/31	17/48
Active MS IFNβ (n = 7)	7/7	7/7	0/7	0/7	-	-
Inactive MS IFNβ (n = 13)	13/13	-	0/13	-	13/13	0/13
Inactive MS NTZ (n = 14)	14/14	0/14	0/14	0/14	14/14	0/14

**Figure 2:**
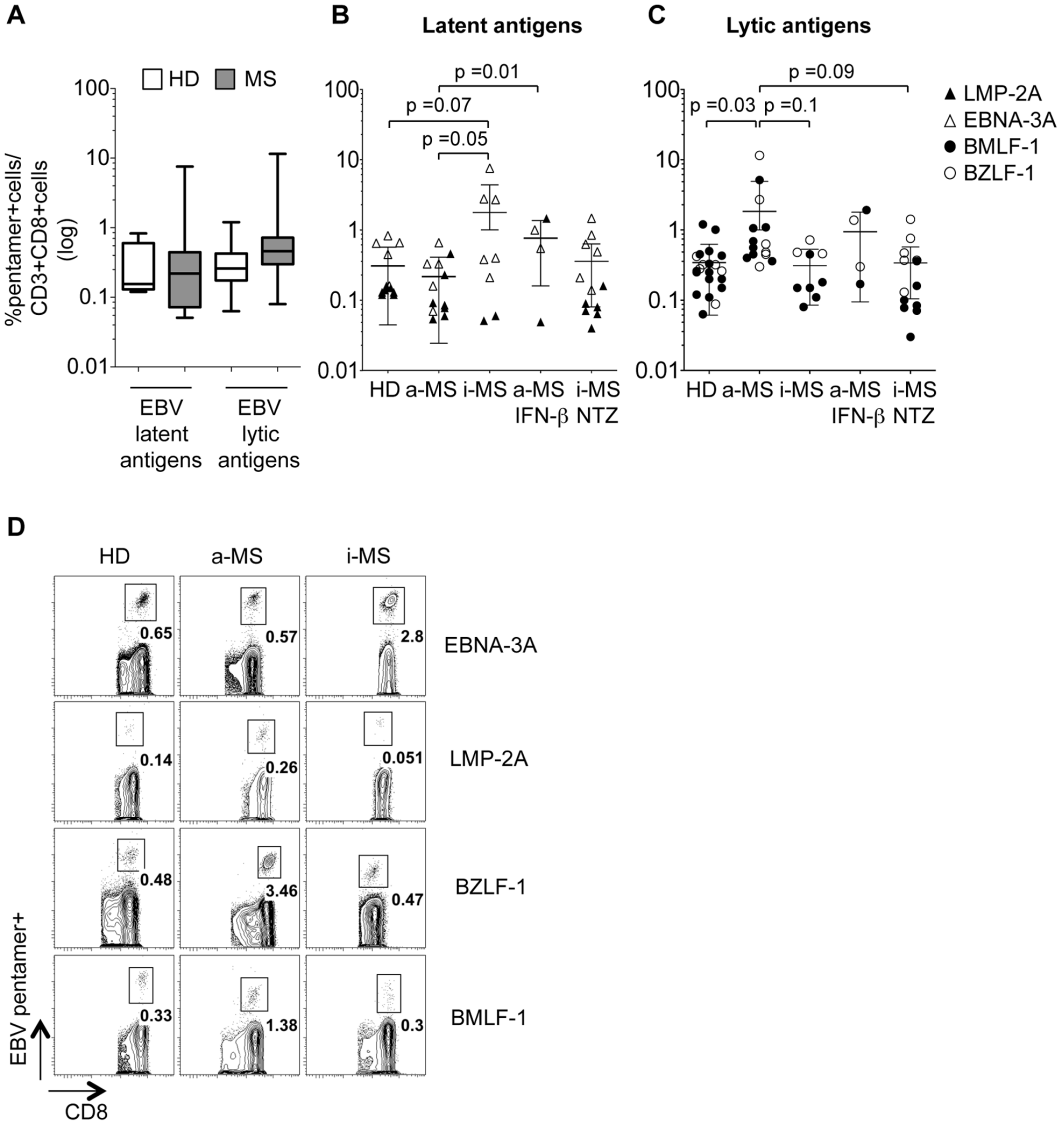
Magnitude of EBV-specific CD8+ T cell responses in HD and MS patients. The frequencies of CD8+ T cells specific for EBV latent and lytic antigens were assessed in HD (n = 17) and total untreated MS patients (n = 26) (A), as well as in untreated active MS (a-MS, n = 13) and inactive MS (i-MS, n = 13) patients, IFN-β-treated active MS patients (a-MS IFN-β, n = 4) and natalizumab (NTZ)-treated inactive MS patients (i-MS NTZ, n = 14) (B, C). The percentages of antigen-specific CD8+ T cells within the CD3+ CD8+ T cell population were analyzed by staining with the corresponding HLA-A*0201 and HLA-B*0801 pentamers. Symbols in B and C refer to individual responses to peptide epitopes from the indicated EBV latent and lytic proteins from each donor. The frequencies were calculated after gating on total, live CD3+ CD8+ T cells. Data are shown in logarithmic scale. In A the bars represent the median ± the minimum and maximum value while in B and C the bars represent mean values ± SD; p values are calculated with unpaired t-test with 95% confidence intervals. (D) Representative flow cytometric expression profiles for pentamer+ CD8+ T cells specific for the indicated EBV latent and lytic antigens in HD, untreated inactive MS and active MS patients are shown in D. Numbers represent percentages of pentamer+ cells within the CD3+ CD8+ T-cell population.

We then evaluated the percentage of pentamer+ CD8+ T cells within the circulating CD3+ CD8+ T cell population in the study subjects with a detectable EBV-specific CD8+ T cell response. Similarly to prevalence, the frequency of latent and lytic antigen-specific CD8+ T cells did not differ significantly between HD (n = 17) and total untreated MS patients (n = 26) (Figure 2 A). Also the frequency of CD8+ T cells specific for EBV and CMV antigens was similar in HLA-A2+ HD and total MS patients (Figure S3A). Again, differences in the immune response to EBV emerged only when patients were stratified according to disease activity (Figure 2 B–D). The frequency of latent antigen-specific CD8+ T cells in inactive MS patients (1.8±2.6%, mean ± SD) tended to be higher than in HD (0.3±0.2%, mean ± SD; p = 0.07) and was significantly higher than in active MS patients (0.22±0.19%, mean ± SD; p = 0.05) (cumulative data and representative plots are shown in Figure 2 B and D, respectively). This difference was mainly due to the fact that 6 out of 8 inactive MS patients recognized EBNA-3A and 3 of these displayed a very strong immune response against this EBV latent antigen (2.7, 2.8 and 7.6% of total circulating CD8+ T cells were EBNA-3A-specific). In contrast, the frequency of lytic antigen-specific CD8+ T cells in active MS patients (1.8±2.8%, mean ± SD) was significantly higher than in HD (0.34±0.28%, mean ± SD; p = 0.03) and tended to be higher than in inactive MS patients (0.3±0.2%, mean ± SD, p = 0.1). The latter difference did not reach statistical significance probably due to the low number of inactive MS patients analyzed (n = 9). These findings therefore indicated more frequent recognition of EBV latent and lytic antigens during inactive and active MS phases, respectively. In contrast, the frequencies of CMV-specific CD8+ T cells did not differ significantly among HLA-A2+ HD, inactive MS and active MS patients (Figure S3B, C). No correlation was found between the frequency of EBV-specific CD8+ T cells and disease duration in inactive and active MS patients (Figure S4).

### Prevalence and frequency of EBV-specific CD8+ T cells of MS patients treated with interferon-β and natalizumab

We then analyzed the EBV-specific CD8+ T cell response in MS patients who were treated with IFN-β (n = 20) and natalizumab (n = 14) (Table 1). IFN-β, the most frequently used first-line treatment for relapsing-remitting MS, has antiviral and immunoregulatory activities and reduces relapse frequency and brain MRI activity in relapsing-remitting MS patients [41]. The monoclonal antibody natalizumab inhibits lymphocyte extravasation into the CNS and is highly effective in suppressing clinical relapses and disease activity in relapsing-remitting MS patients who fail to respond to first-line therapies [42].

Among 20 MS patients treated with IFN-β for 1 to 11 years (median = 4 years), 7 and 13 were in the active and inactive phase of disease, respectively (Table 2). The prevalence of the CD8+ T cell response to EBV latent and lytic antigens was similar in total untreated and IFN-β-treated MS patients (Figure 1 A, B). However, none of the 13 IFN-β-treated inactive patients had a detectable CD8+ T cell response to EBV (Figure 1 B) and CD8+ T cells for CMV were found only in 1 of 9 HLA-A02+ IFN-β-treated inactive patients (data not shown), indicating that effective IFN-β therapy is associated with a general inhibition of the antiviral response. In contrast, 57% (4/7) and 66% (4/6) of the IFN-β-treated MS patients with active disease had a detectable CD8+ T cell response to EBV (Figure 1 B) and CMV (data not shown), respectively. Moreover, the frequency of CD8+ T cells specific for EBV latent, but not lytic, antigens was significantly higher in IFN-β-treated (0.8±0.6%, mean ± SD) than in untreated (0.2±0.2%, mean ± SD ; p = 0.01) active MS patients (Figure 2 B, C).

After 8 to 16 months (median = 12 months) of treatment with natalizumab, all 14 MS patients analyzed were in the inactive phase of disease, both clinically and by MRI (Table 2). Unexpectedly, nearly all patients in this group had a detectable CD8+ T cell response to EBV latent and lytic antigens (87% and 93%, respectively) (Figure 1 B). This prevalence was significantly higher than that found in HD and any other group of MS patients (p<0.001). Nevertheless, the frequencies of latent and lytic antigen-specific CD8+ T cells in natalizumab-treated MS patients were similar to those found in untreated inactive MS patients (Figure 2 B, C). As expected for patients in the inactive phase of disease, the frequency of lytic antigen-specific CD8+ T cells tended to be lower in natalizumab-treated patients than in untreated active MS patients (p = 0.09) (Figure 2 C). The frequency of CMV-specific CD8+ T cells did not differ among HD, untreated and natalizumab-treated MS patients (data not shown).

### Longitudinal study of EBV-specific CD8+ T cell responses

**Figure 3:**
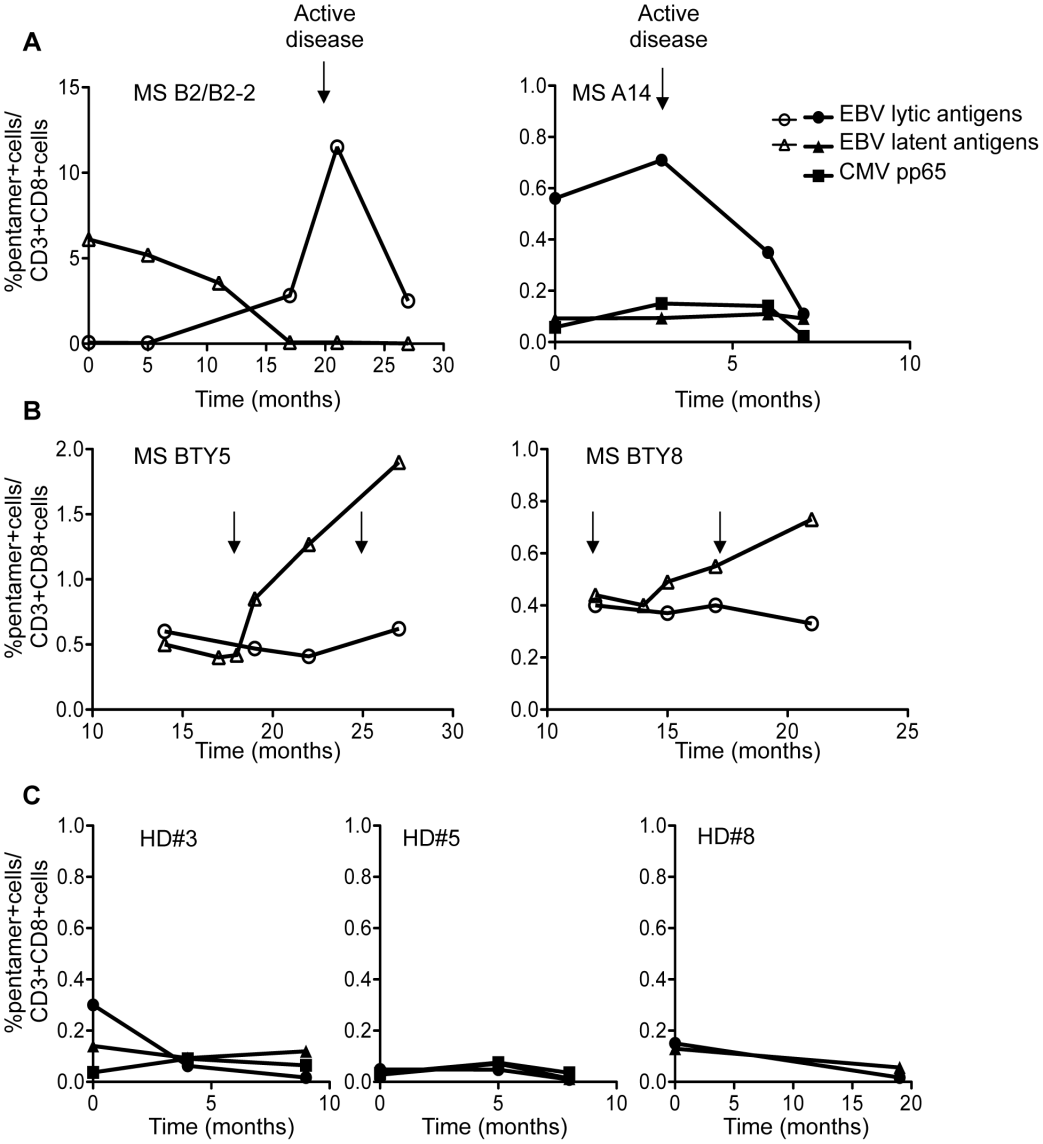
Longitudinal monitoring of EBV-specific CD8 T cell responses in relapsing remitting MS. The frequencies of CD8+ T cells specific for EBV lytic and latent antigens and CMV pp65 antigen were measured at different time points in 2 untreated MS patients (A), 2 natalizumab-treated MS patients (B) and 3 HD (C) using HLA-A*0201 and HLA-B*0801 pentamers. All patients were monitored clinically and with MRI at the times indicated by the arrows. The untreated MS patients had gadolinium-enhancing MRI lesions 20 days (MSB2/B2-2) and 1 day (MSA14) before the peak of the CD8+ T cell response to EBV lytic antigens while the 2 natalizumab-treated patients (MS BTY5 and MS BTY8) did not show any disease activity. Percentages of pentamer^+^ cells within the CD3+ CD8+ population are given on the y axis; the numbers on the x-axis indicate months since the start of the observation period (A, C) or of drug therapy (B).

We then asked whether changes in the EBV-specific CD8+ T cell response during active and inactive MS phases could be detected in longitudinal studies. Despite experiencing clinical relapses, two patients (HLA-B08+ B2/B2-2 and HLA-A02+ A14) in our cohort refused immunomodulatory therapy and agreed to be monitored periodically for 27 and 7 months, respectively. Patient B2/B2-2, who was clinically silent and was diagnosed MS one year before our analysis started, displayed a very highy frequency of EBNA-3A-specific CD8+ T cells (6% of circulating CD8+ T cells) at the beginning of the observation period (month 0; Figure 3 A). EBNA-3A-specific CD8+ T cells progressively decreased during the subsequent months and became undetectable between month 17 and 27. In parallel, the frequency of BZLF-1 specific CD8+ T cells, which were undetectable at previous time points, abruptly increased and reached a peak (11% of total circulating CD8+ T cells) at month 21 in concomitance with the presence of active MRI lesions. The percentage of BZLF-1-specific CD8+ T cells in the CD8+ T cell population then declined to 2.5% in the subsequent 6 months, denoting marked expansion and subsequent contraction of the immune response toward this EBV lytic antigen (Figure 3 A).

In patient A14, who experienced frequent clinical relapses, the frequency of CD8+ T cells specific for the EBV lytic antigen BMLF-1 ranged between 0.56 and 0.71% during a clinical relapse (months 0 and 3 of the observation period) and in the presence of an active MRI scan, and dropped to 0.11% in the subsequent 4 months (Figure 3 A). During the same period, the frequency of CD8+ T cells specific for the EBV latent antigen LMP-2A and for CMV pp65 antigen remained low and stable (Figure 3 A).

Longitudinal analysis of EBV-specific CD8+ T-cell responses was also performed in 2 HLA-B08+ MS patients (BTY5 and BTY8) treated with natalizumab and monitored periodically for 15 and 12 months, respectively, starting at 12–14 months after therapy initiation. Both natalizumab-treated patients were in the inactive phase of disease (according to clinical and MRI evaluation) and had a detectable CD8+ T cell response to EBNA-3A and BZLF-1 (Figure 3 B). However, while the frequency of BZLF-1-specific CD8+ T cells was stable during the whole observation period a steady rise in the CD8+ T cell response to EBNA-3A was observed in both patients after 15–18 months of therapy (Figure 3B). Three HD followed for 8 to 19 months did not show any significant variation in the frequency of CD8+ T cells specific for EBV latent and lytic antigens (Figure 3 C).

### BZLF-1 expression in the inflamed MS brain

The increased frequency of CD8+ T cells specific for two EBV lytic antigens (BZLF-1, BMLF-1) in the blood of MS patients with an active MRI scan indirectly suggests a response to a previous or concomitant viral reactivation in the brain. In search for a link between immunological findings and brain inflammation, we examined the expression of BZLF-1 mRNA and protein in post-mortem brain tissue from 7 patients who died in the secondary progressive phase of MS and were characterized by a severe clinical course and substantial brain inflammation. The MS brain samples selected for this study contained immunologically active (both active and chronic active) lesions in the white matter and highly inflamed meninges with B-cell follicle-like structures that we previously showed to be major EBV reservoirs [32]–[34].

**Figure 4:**
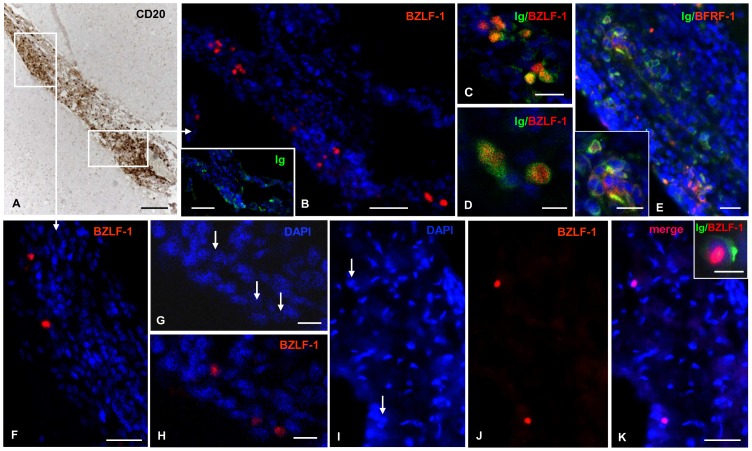
Immunohistochemical detection of BZLF-1 in the inflamed meninges of the MS brain. (A) A prominent immune infiltrate comprising a perivascular B-cell-follicle-like structure is visualized with anti-CD20 antibody in the meninges lining a cerebral sulcus of a MS case. (B) Immunostaining for the EBV immediate early lytic protein BZLF-1 in a serial section reveals the presence of several BZLF-1+ cells at the periphery of the B-cell follicle. Ig+ cells are present in the same area (inset). (C, D) High magnification pictures of Ig+ plasmablasts/plasma cells (green) coexpressing BZLF1 (red); these groups of cells correspond to those shown in the top left and bottom right parts of panel B. (E) Expression of the EBV early lytic protein BRFR1 (red) in a substantial proportion of Ig+ (red) plasmablasts/plasma cells in the same area shown in panel B; double immunofluorescence staining for Ig and BFRF-1 was performed in a section adjacent to those stained in A and B. (F) Two BZLF-1+ cells in another part of the B-cell-rich infiltrate shown in panel A. Nuclei were stained with DAPI (blue) in panels B-F. (G-K) Other examples of BZLF-1+ cells at the border of a meningeal B-cell follicle (G, H) and in a diffuse meningeal infiltrate (I-K) from a different MS case. Arrows in G and I point to the nuclei that are immunoreactive for BZLF-1 in H and J, respectively. The inset in panel K shows an Ig+ cell (green) with a BZLF-1+ nucleus (red) at high magnification. Bars = 100 µm in A and inset in B; 50 µm in B; 20 µm in C, E, F, K and inset in E; 10 µm in D, G, H and inset in K.

**Figure 5:**
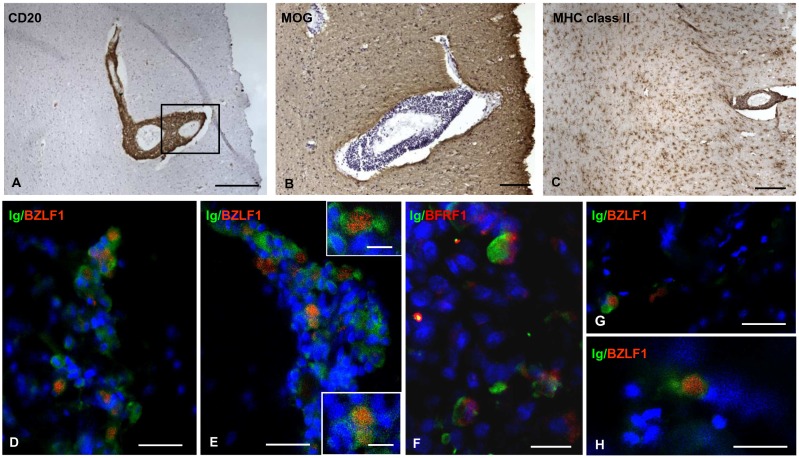
Immunohistochemical detection of BZLF-1 in acute white matter lesions of the MS brain. (A) A large, B-cell enriched perivascular immune infiltrate surrounding 3 blood vessels in an active white matter lesion of a MS case is visualized with anti-CD20 antibody. (B) Immunostaining for myelin-oligodendrocyte glycoprotein (MOG) in a serial section reveals presence of myelin. (C) The same lesion shows massive microglia/macrophage activation in the parenchyma. Sections shown in panels A–C were counterstained with hematoxylin. (D, E) Double immunofluorescence staining for Ig (green) and BZLF-1 (red) reveals the presence of several Ig+ plasmablasts/plasma cells co-expressing BZLF-1 in the portion of the perivascular cuff marked with a frame in panel A. The upper and lower insets in E show 1 and 2 Ig+ cells co-expressing BZLF-1 at high power magnification, respectively. (F) Double immunofluorescence staining for BFRF-1 (red) and Ig (green) shows presence of double labelled BFRF-1+/Ig+ cells in the same area of the perivascular cuff stained for BZLF-1 in E. (G, H) Cells double stained for Ig (green) and BZLF-1 (red) in smaller perivascular cuffs of the same active lesion shown in A–C. Bars: 200 µm in A and C; 100 µm in B; 50 µm in D, E, G; 20 µm in F and H; 10 µm in the insets in D.

In preliminary experiments aimed at evaluating the specificity and binding of anti-BZLF-1 monoclonal antibody, we observed BZLF-1 immunoreactivity in the nucleus of EBV transformed B95-8 cells and in an EBV+ tonsil from a patient with infectious mononucleosis (Figure S5). Conversely, no BZLF-1+ cells were detected in sections of a non pathological human brain, of a brain from a patient with tuberculous meningoencephalitis and of an EBV-negative lymphoma (Figure S5). BZLF-1 immunoreactivity was detected in brain samples of all MS cases analyzed using immunohistochemical techniques (n = 5). Both isolated and small groups of BZLF-1+ cells were present in the inflamed meninges, at the periphery of B-cell follicle-like structures (Figure 4 A–H) and in diffuse inflammatory cell infiltrates (Figure 4 I–K). BZLF-1+ cells were also present in the perivascular cuffs of inflamed blood vessels in active white matter lesions characterized by a high density of intraparenchymal foamy macrophages (Figure 5), but not in demyelinated, chronic active and inactive white matter lesions (data not shown), thus linking EBV reactivation to acute inflammation. Nearly all BZLF-1+ cells infiltrating the meninges (Figure 4 C, D, K) and active WM lesions (Figure 5 D, E, G, H) were identified as Ig-producing plasmablasts/plasma cells, which is consistent with the knowledge that EBV reactivates upon B-cell differentiation into plasma cells [43]. At these sites the proportion of Ig+ cells expressing BZLF-1 ranged between 1 and 10%. BZLF-1+ plasma cells were detected in the same infiltrated brain areas where plasma cells expressing BFRF1, an EBV early lytic protein induced by BZLF-1 [44], were also found (Figure 4 E, Figure 5 F).

**Figure 6:**
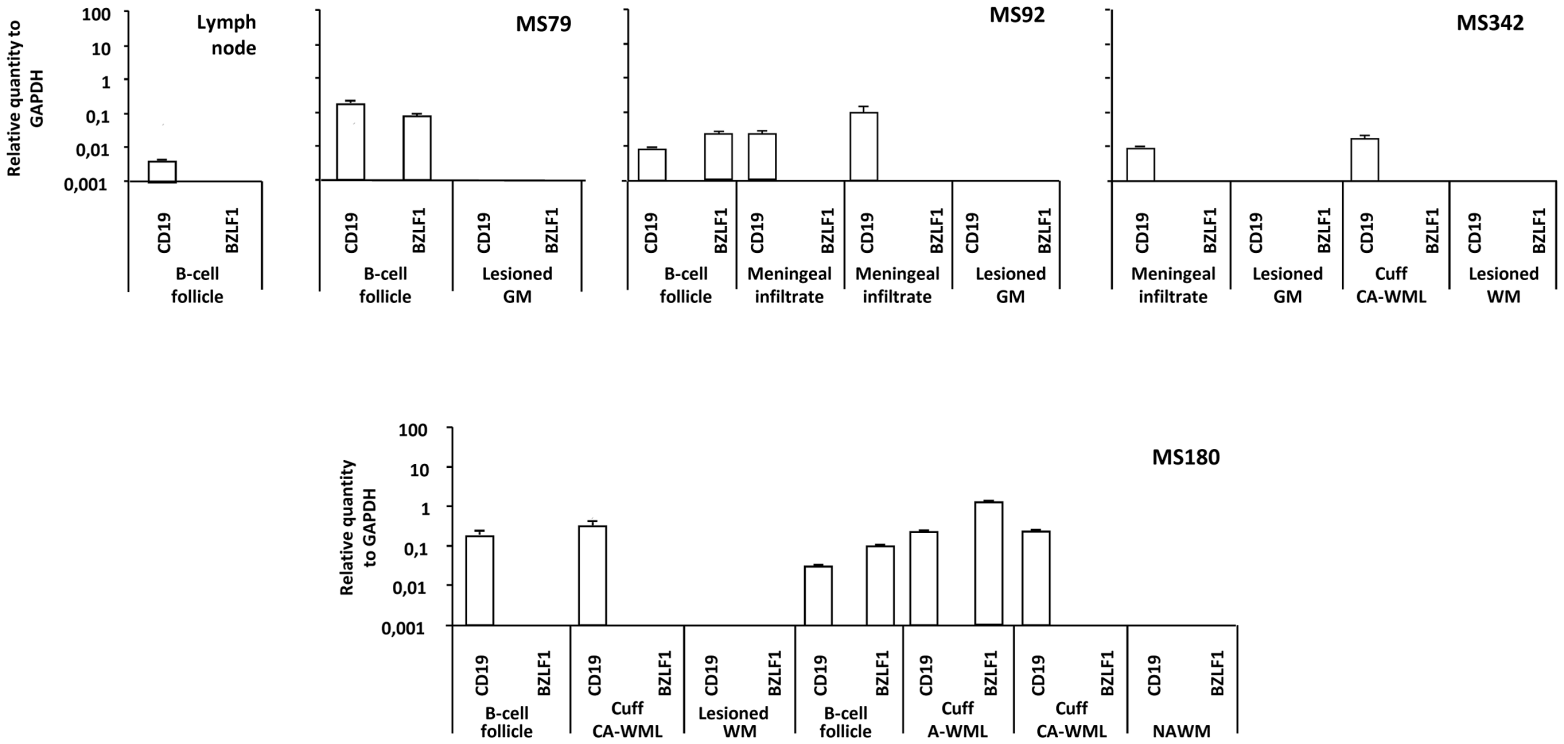
BZLF-1 gene expression in MS brain immune infiltrates. RNA was extracted from the indicated areas microdissected from sections of a control lymph node and from brain sections of 4 MS cases (MS79, MS92, MS342, MS180). Quantitative real-time RT-PCR for BZLF-1, for CD19 and GAPDH was performed following pre-amplification of cDNAs, as described in Materials and methods. Data for BZLF-1 and CD19 are normalized for the GAPDH level of expression. BZLF-1 mRNA was detected in 3 out of 4 meningeal B-cell follicles and in the perivascular cuff of an active white matter lesion (cuff A-WML), but not in lymph node, perivascular cuffs of chronic active WM lesions (cuff CA-WML), demyelinated white matter parenchyma (lesioned WM), demyelinated grey matter parenchyma (lesioned GM), and normal-appearing white matter (NAWM). The presence of CD19 signal indicates that the microdissected region includes a B-cell containing immune infiltrate. Values are means ± SD of triplicate values.

Expression of BZLF-1 was also investigated using quantitative real-time RT-PCR in 4 inflamed MS brain samples, 2 of which had been analyzed by immunohistochemistry. No BZLF-1 RNA was detected in whole MS brain sections (data not shown). This negative result was expected given the paucity of EBV lytically infected plasma cells relatively to the large and heterogeneous cell population of the MS brain. To enrich for EBV infected cells and increase the sensitivity of the technique, perivascular and meningeal inflammatory cell infiltrates and the surrounding, non infiltrated brain parenchymal regions were harvested from MS brain sections using laser capture microdissection and analyzed using pre-amplification, quantitative real time RT-PCR for BZLF-1. CD19 transcripts were also analyzed to optimally discriminate between infiltrated and non infiltrated brain areas. BZLF-1 transcripts were detected in the perivascular cuffs isolated from one active MS lesion and in 3 out of 4 meningeal B-cell follicles but not in 3 chronic active lesions, 4 sparse meningeal infiltrates and 7 non infiltrated parenchymal regions (Figure 6). A control lymph node was negative for BZLF-1. Thus, both immunohistochemical and RT-PCR findings corroborated BZLF-1 expression and therefore shift to EBV lytic infection in immunologically active white matter lesions and ectopic B-cell follicles.

### EBV lytically infected cells in the MS brain are targeted by a cytotoxic immune response

**Figure 7:**
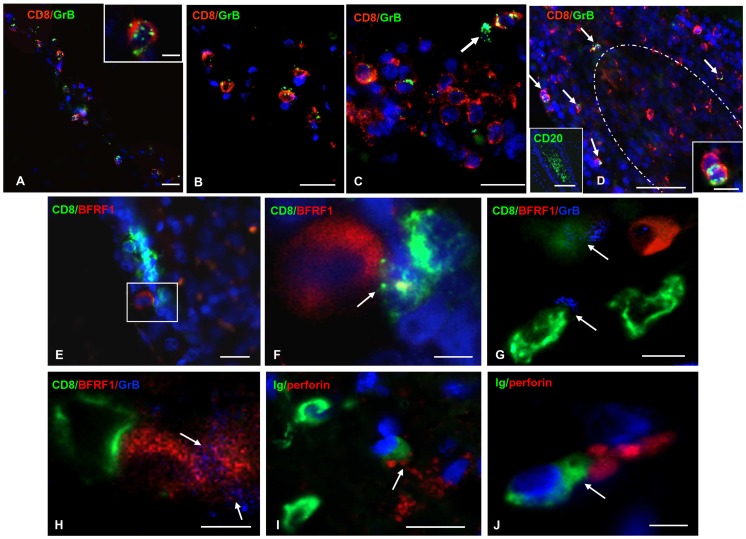
Cytotoxic attack on EBV infected cells in the MS brain. (A, B) Double immunofluorescence stainings for granzyme B (GrB, green) and CD8 (red) performed on sections of highly inflamed MS brain samples show granzyme B expression in a substantial proportion of CD8+ T cells present in two perivascular inflammatory cell infiltrates of an active white matter lesion; granzyme B-containing granules are localized in the cytoplasm and on the surface of CD8+ cells (inset in A). (C) Presence of granzyme+ CD8+ cells in a diffuse meningeal immune infiltrate. The arrow points to one of the rare granzyme B+/CD8− cells. (D) Double immunofluorescence staining for CD8 (red) and granzyme B (green) shows that granzyme B−/CD8+ cells accumulate outside and inside a B-cell follicle (marked with a dotted line and stained for CD20 in the left inset), whereas double labelled granzyme B+/CD8+ cells are detectable only at the periphery the B-cell rich area (arrows). Two CD8+ cells, one of which is also granzyme B+, are shown at high power magnification in the inset on the right. (E) Group of CD8+ cells (green) close to an EBV lytically infected, BFRF-1+ cell (red) at the border of a B-cell follicle. (F) The CD8+ cell closer to the BRFR-1+ cell extends cytoplasmic processes (arrow) that adhere to the infected cell (high magnification of the area marked with the frame in E). (G) Three CD8+ cells (green) with a lymphobast-like morphology are close to, but do not touch, a BFRF-1+ cell (red) in the inflamed meninges; two of the CD8+ cells display granzyme B-containing granules on their surface (blue, arrows). (H) Two BFRF-1+ cells (red), one of which is enclosed by a CD8+ cell (green) and both are covered by granzyme B+ granules (blue, arrows) at the border of a meningeal B-cell follicle. (I, J) Double immunofluorescence stainings for perforin (red) and Ig (green) show perforin granules polarized toward Ig+ plasma cells (arrows) in active white matter lesions. Nuclei are stained with DAPI (blue) (A, B, C, D, E, F, I, J). Bars : 100 µm in the left inset in D; 50 µm in D; 20 µm in A–C, E, I; 10 µm in G, H and right inset in D; 5 µm in F, J and inset in A.

Then, we searched for interactions between cytotoxic CD8+ T cells and EBV infected cells in the same MS brain samples in which BZLF-1 protein and/or RNA were detected. We first analyzed the presence and frequency of granzyme B-expressing CD8+ T cells and their relationship to EBV litically infected cells. We observed that most granzyme B+ cells in the MS brain co-expressed CD8 and that the fraction of CD8+ T cells expressing granzyme B ranged between 5 and 60% in the different MS cases and brain areas analyzed, the highest values being detected in the perivascular cuffs of active white matter lesions (Figure 7 A, B). In the meninges, granzyme B+/CD8+ T cells were present in diffuse meningeal infiltrates and at the periphery of B-cell follicle-like structures, but were rarely seen inside these structures (Figure 7 C, D).

Given the nuclear localization of BZLF-1 and the relatively small number of BZLF-1+ cells in the MS brain it was extremely difficult to see contacts between granzyme B+ cells and lytically infected cells using double immunofluorescence for BZLF-1 and CD8 or granzyme B. We therefore stained MS brain sections for the EBV lytic protein BFRF1 which has a perinuclear localization and has been detected in a higher fraction of plasma cells (up to 50%) compared to BZLF-1 [32]. We observed lymphoblastoid CD8+ T cells adhering to or secreting granzyme B toward BFRF1+ cells as well as contacts between CD8+ T cells and BFRF1+ cells displaying granzyme B immunoreactivity on their surface (Figure 7 E–H). Such cytotoxic contacts were frequently observed in sparse meningeal infiltrates and active white matter lesions, but not inside ectopic B-cell follicle-like structures. Finally, staining of MS brain sections for perforin and Ig allowed to visualize perforin granules polarized toward Ig+ cells inside the perivascular cuffs of active white matter lesions (Figure 7 I, J), supporting further the idea that EBV harbouring cells might be the target of a cytotoxic attack.

## Discussion

Altered control of EBV infection in individuals susceptible to MS is suspected to play a role in the development of immune dysfunction causing CNS pathology [3]–[5]. Higher serum titers of EBNA-1 IgG are associated with an increased risk of MS [15], increased conversion from a clinically isolated syndrome to definite MS [16] and more severe disease activity and clinical progression [16], [36]. Virus-specific CD8+ T cell responses play an essential role in the control of EBV infection [45] and have been investigated in previous studies in MS using mainly IFN-γ ELISPOT analysis in PBMCs stimulated in vitro with EBV+ lymphoblastoid cells [20], [22], viral lysates [21], individual [19] or pooled [21] EBV lytic and latent peptides, and more recently using MHC-peptide tetramer staining [23]. Several studies have shown that EBV-specific CD8+ T cell responses are significantly higher in MS than in HD or in patients with other inflammatory neurological diseases [19]–[21]. However, lack of significant differences between MS patients and controls [16], [23] and even reduced frequency of EBV-specific CD8+ T cells in MS patients [22] have also been reported. Use of cryopreserved versus freshly isolated PBMCs, analysis of patients with relapsing-remitting and progressive MS courses, and lack of stratification of patients according to disease activity may have hampered a clear understanding of the possible link between EBV-specific CD8+ T cell responses and MS pathogenesis.

To obtain an accurate pattern of CD8+ T cell *in vivo* specificities, we have used highly standardized flow cytometric analysis with EBV-specific pentamers, that unequivocally identify antigen-specific CD8+ T cells, on freshly isolated PBMCs obtained from HD and relapsing-remitting MS patients. Importantly, both untreated and treated MS patients were studied and disease activity was evaluated in most patients with gadolinium-enhanced MRI shortly before or at the time of blood collection. Such a rigorous selection justifies the relatively small number of MS patients analyzed.

The first main finding of this study is that differences in the prevalence and magnitude of the CD8+ T cell response to certain EBV latent and lytic proteins between MS patients and HD and within the MS cohort emerge only when patients are stratified according to disease activity and inactivity. By showing that fewer inactive MS patients have a detectable CD8+ T cell response against EBV lytic antigens compared with HD and active MS patients and that the frequency of lytic antigen-specific CD8+ T cells is higher in active MS patients than in HD and inactive MS patients, we demonstrate for the first time that changes in the immune control of EBV replication are associated with the active and inactive phases of MS. This is corroborated by the longitudinal study performed in two untreated MS patients showing a peak in the frequency of CD8+ T cells to EBV lytic antigens during active disease. Of the two EBV lytic antigens analyzed, BZLF-1 is a transactivator expressed at the very initiation of the lytic cycle and is involved in the induction of early lytic proteins, including BMLF-1 [6]. Thus, an increase in BZLF-1- and BMLF-1-specific CD8+ T cells in concomitance with acute brain inflammation on MRI strongly suggests an attempt of the immune system to control intracerebral foci of EBV replication. On the other hand, a logical explanation for the decrease in lytic antigen-specific T cells associated with inactive MS could be elimination of lytically infected cells brought about by the strong cytotoxic response occurring in the active disease phase. In this context, it is important to recall that EBV DNA load in the blood of MS patients does not differ significantly from that in healthy EBV carriers [16], [18], [23] indicating that fluctuations in EBV-specific CD8+ T cell responses in MS patients do not result in a generalized impairment of the immune control of EBV infection. In contrast with the present findings, Jilek et al. [23] did not observe differences in the prevalence and frequency of CD8+ T cell responses to BZLF-1 and BMLF-1 between MS patients and control subjects. However, this study was not restricted to patients with relapsing-remitting MS, did not distinguish between patients with active and inactive disease and used cryopreserved PBMCs. Importantly, in our study both the prevalence and magnitude of the CD8+ T cell response to CMV were similar in HD and untreated MS patients, irrespective of disease activity, indicating that the differences observed in EBV-specific immunity are not the consequence of a general activation of antiviral immune responses driven by a still unknown MS-associated immune dysfunction.

Despite the fact that the prevalence of the CD8+ T cell response to EBV latent antigens in inactive MS patients was similar to that in HD and tended to be lower than in active MS patients, we found that the magnitude of the CD8+ T cell response to EBNA-3A was higher in inactive MS patients than in HD and active MS patients. Very high numbers of EBNA-3A-specific CD8+ T cells were detected in half of the inactive MS patients harbouring this immune reactivity (2.7 to 7.6% of the circulating CD8+ T cells versus <1% in HD and active MS patients). Furthermore, longitudinal monitoring of a patient with a recent diagnosis of MS showed substantial reduction of EBNA-3A-specific CD8+ T cells just before the active disease phase and the rise of lytic antigen-specific CD8+ T cells. It is known that EBNA-3A is expressed shortly after EBV infection of B cells together with the whole set of EBV latent proteins (EBNA-LP, −1, −2, 3A, 3B, and −3C, LMP-1, LMP-2A, LMP-2B) that are essential to drive infected B cells into proliferation (latency III or growth program) [6] and elicit strong T-cell responses [45]. Most EBV-encoded latent antigens, including EBNA-3A, are then sequentially downregulated as EBV establishes a persistent infection in memory B cells (latency II and I programmes) [6]. Thus, the study of EBNA-3A-specific CD8+ T cells suggests that at least in some inactive MS patients there is an attempt by CD8+ T cells to control abnormal expansion of a latently infected B-cell pool. A decrease in the immune response to EBNA-3A could reflect a change in EBV latency programme and, possibly, set the stage for switching to the lytic cycle. Of interest, more abundant EBNA-3A-specific CD8+ T cells were detected only in MS patients with a short disease duration (<5 years). A decrease in immune reactivity toward EBNA-3A with disease progression could be due to reduced antigenic stimulation or to T-cell exhaustion which is known to occur during uncontrolled, chronic viral infections [46]. Relevant to this, we have shown that most EBV latently infected B cells accumulating in the inflamed MS brain during late-stage disease are memory B cells expressing the latency II programme [32], [33].

Based on the present findings, we propose that failure to fully control EBV latent infection in an immune privileged site like the CNS could lead to recrudescence of EBV reactivation. Exposure to newly synthesized viral antigens would promote expansion of lytic antigen-specific CD8+ T-cells targeting intracerebral viral deposits and hence inducing the active phase of MS. Of relevance for the present findings, it has been shown that after primary EBV infection and during establishment of EBV persistence CD8+ T cells specific for some EBV epitopes disappear from the circulation after having upregulated Programmed Death-1 (PD-1) inhibitory receptor, probably as a consequence of inadequate antigenic stimulation [47]. We are currently evaluating whether fluctuations in EBV-specific CD8+ T cells in relapsing-remitting MS might be associated with changes in PD-1 expression levels and T-cell function (i.e., cytokine profile and cytotoxic activity). It would be also interesting to compare the quality of the CD8+ T cell response to EBV in MS with that in systemic lupus erythematosus, an autoimmune disease associated with marked systemic EBV dysregulation [48] and impaired cytotoxic immune response to the virus [49].

The second main finding of this study is that treatment of relapsing-remitting MS patients with IFN-β and natalizumab is associated with marked changes in the CD8+ T cell response to viral antigens. We have shown that CD8+ T cells specific for EBV latent and lytic antigens and for CMV antigen were detectable in a substantial fraction of the patients entering active disease despite IFN-β treatment, but in none, except one, of the IFN-β-treated patients with inactive disease. It is likely that such a strong reduction in the CD8+ T cell response to both viruses might due to the direct antiviral activity of the drug [41]. Recently, Comabella et al. [50] reported that clinically effective IFN-β therapy is associated with downregulation of proliferative T cell responses to EBNA-1 without significant changes in the CD8+ T cell response against other (pooled) EBV antigens of the latent and lytic phase. Discrepancies with the present study could be due to technical issues, as discussed above. We have also shown that most natalizumab-treated MS patients, all of which were in the inactive phase of disease, had a detectable CD8+ T cell response to EBV. Such high prevalences could be related to the fact that natalizumab treatment causes a marked increase in lymphocyte numbers in the blood due to interference with lymphocyte extravasation and trafficking in lymphoid and non-lymphoid tissues [51]. Importantly, we found that in natalizumab-treated MS patients the frequency of CD8+ T cells specific for EBV lytic antigens was similar to that in HD and untreated inactive MS patients and stable over time (up to 22–27 months of therapy). It therefore seems significant that the present analysis, though limited to a relatively small number of donors, consistently showed no expansion of EBV lytic antigen-specific CD8+ T cells during the inactive phase of MS regardless of presence or absence of therapy. In contrast, the frequency of EBNA-3A-specific CD8+ T cells, which was comparable in untreated and natalizumab-treated inactive MS patients within 8–16 months of therapy, progressively increased during the second year of therapy in 2 longitudinally monitored patients. These observations suggest dysregulation of EBV latent infection upon prolonged treatment with natalizumab. Although further studies are needed to clarify these aspects, analysis of EBV-specific CD8+ T cell responses in MS patients may help identify biomarkers useful for therapy monitoring and shed light into the mechanisms underlying drug efficacy.

The third main finding of this study is that BZLF-1, one of the two EBV lytic proteins recognized by CD8+ T cells expanding in the blood of active MS patients, is expressed in post-mortem MS brains with prominent immune infiltrates. The demonstration of BZLF-1 protein and RNA in active white matter lesions, which likely correspond to gadolinium-enhanced MRI lesions, and in the inflamed meninges, where changes in water content cannot be detected on MRI, lends support to the idea that acute brain inflammation in MS is associated with switch to the viral lytic cycle. In line with our previous results [32], we have also shown that in the MS brain EBV reactivates in plasma cells and that the latter can be found in close contact with lymphoblastoid CD8+ T cells producing cytolotic enzymes. However, absence of CD8+ granzyme B+ T cells inside meningeal B-cell follicles, which contain a high frequency of EBV latently infected cells [32]–[34], suggests that a local suppressive environment created by the virus itself to elude immune control [52] could hamper virus clearance from these structures. A cytotoxic attack toward EBV infected cells in the MS brain is consistent with enrichment in EBV-specific CD8+ T cells in the cerebrospinal fluid (CSF) of patients with early MS [53], with increased CSF levels of granzymes during relapse in relapsing-remitting MS patients [54], and with preferential expansion of CD8+ T cells in MS brain lesions and CSF [55], [56].

Defects in the control of viral infections are suspected to promote the development of autoimmune diseases [57]. Nearly all of the genes whose variants have been associated with the risk of developing MS are implicated in immune system function [2], making it plausible that in susceptible individuals subtle differences in the regulation of the immune response might allow an EBV infection to be established in the CNS and become the target of an immunopathological response. Experimental studies suggest that upon infection with persistent viruses establishment of extralymphatic viral sanctuaries depends both on organ anatomy and defective synergies between CD8+ T-cell- and antibody-mediated immune responses [58]. The present results do not answer the question of whether EBV dysregulation is consequence or cause of MS but disclose a link between EBV reactivation, antiviral immune response and disease activity during the relapsing-remitting stage of MS. Such a scenario is consistent with the results of randomized, double-blind, placebo-controlled clinical and MRI studies of anti-herpesvirus therapy in relapsing-remitting MS showing that anti-herpesvirus drugs inhibiting viral replication have beneficial effects in subgroups of patients with higher exacerbation rates and more severe disease activity [59], [60]. Further work is needed to better understand whether and how an altered balance between EBV and the host immune system contributes to MS onset and verify the potential benefits of new antiviral drugs in controlling MS [61].

## Materials and Methods

### Ethics statement

All blood samples were obtained following acquisition of the study participants' written informed consent. The study protocol was reviewed and approved by the local ethics committes of S. Camillo Forlanini Hospital, Tor Vergata University, S. Andrea Hospital, and Fondazione S. Lucia. Use of post-mortem human brain material for the study purposes has been approved by the ethics committee of the Istituto Superiore di Sanità.

### Patients and healthy donors

MS patients and HD were recruited between 2008 and 2012 from Tor Vergata University, S. Camillo Forlanini and S. Andrea Hospitals in Rome. We enrolled 250 patients who were diagnosed the relapsing-remitting form of MS according to the 2005 revised McDonald's criteria [62]. A neurologist (SR, CG, FB, DC, MS) examined the patients, including assessment of the EDSS and confirmation of clinical relapse or remission. Of the 250 enrolled subjects, 113 MS patients were selected for this study according to their HLA genotype (HLA-A*02101, B*0801) for which well characterized EBV and CMV peptide antigens have been described [38]–[40]. Seventy-nine patients were free of therapy for at least 3 months, 20 patients were treated with IFN-β subcutaneously (12 with IFN-β 1a and 8 with IFN-β 1b) for 1–11 years (median = 4 years) and 14 MS patients were treated with natalizumab for 8–16 months (median = 12 months). The control subject group included 43 HD matched for sex and age and selected for their HLA genotype (HLA-A*02101, B*0801). The demographic and clinical characteristics of HD and MS patients are summarized in Table 1. At the time of peripheral blood collection 38 and 75 MS patients were in the active and inactive phase of the disease, respectively, based on clinical assessment and brain MRI (Table 2). Four MS patients (2 untreated and 2 treated with natalizumab) and 3 HD were monitored longitudinally for 7–27 months and blood was drawn every 3 to 6 months.

Seventy-six % (60/79) of untreated patients and all IFN-β-treated patients were examined by brain MRI with gadolinium enhancement on the same day or within 1 week before blood collection; only in one case MRI was performed 3 weeks before blood collection. All natalizumab-treated patients were monitored with MRI every 6 months. Acquisition of brain MRI scans was obtained in a single session. Conventional MRI scans were acquired including the following sequences: Fast Fluid Attenuated Inversion Recovery (FLAIR), T1 weighted images (T1-WI) before and after gadolinium administration covering the whole brain. The gadolinium enhanced T1-WI scans were obtained for all patients 15 minutes after admnistration of gadolinium (0,1 mmol/kg). MRI scans were classified as active if there was at least one gadolinium enhancing lesion. As shown in Table 2, the majority (86%) of active MS patients included in this study had both clinical manifestations and an active MRI scan, while a minority showed either clinical (6%) or MRI (8%) evidence of disease activity. Conversely, all inactive patients exhibited neither clinical manifestations nor disease activity on MRI.

### Flow cytometry with antibodies and peptide/MHC pentamers

PBMCs were isolated on a Ficoll gradient (Ficoll-Paque PLUS, GE Healthcare) and stained with pre-titrated antibodies. To evaluate the CD8+ T cell response to EBV latent and lytic antigens, PBMC from MS patients and HD were stained with fluorochrome-labeled pentamers (ProImmune, Oxford, UK). The analysis was conducted on freshly isolated PBMC with the exclusion of dead cells, providing an accurate pattern of CD8+ T cell *in vivo* specificities. We analyzed CD8+ T cells specific for two EBV lytic protein epitopes, the HLA-A*0201 restricted epitope (GLCTLVAML) from BMLF-1 and the HLA-B*0801 restricted epitope (RAKFKQLL) from BZLF-1, and for two EBV latent protein epitopes, the HLA-A*0201 restricted epitope (CLGGLLTMV) from LMP-2A and the HLA-B*0801 restricted epitope (FLRGRAYGL) from EBNA-3A. The CD8+ T cell response to an HLA-A*0201 restricted immunodominant peptide (NLVPMVATV) from pp65 of human cytomegalovirus (CMV) was studied as a control for anti-viral MHC class I restricted CD8 T-cell responses.

One x 10^6^ PBMCs were stained with 10 µl of PE conjugated-pentamers alone, washed with PBS and then stained with anti human CD3 APC Alexa e780 (eBioscience Inc., San Diego, CA) and CD8 ECD (Beckman Coulter, Brea, CA). Cells were also stained for dead cell exclusion (Fixable Dead Cell Stain Kits, Invitrogen, Life Technologies, Paisley, UK). The samples were acquired on a CyAN ADP cytometer (Beckman Coulter) and analysed by FlowJo software (Tree Star, Ashland, OR). Frequencies of pentamer+ cells below 0.02% of CD3+ T cells were considered as background staining as indicated by the manufacturer. An example of the gating strategy used to identify pentamer^+^ cells is shown in Figure S1.

### Patients and tissues for immunohistochemical and molecular studies

Thirteen cerebral tissue blocks from 7 MS cases (MS79, MS92, MS121, MS154, MS180, MS234, MS342) who died in the secondary progressive phase of MS and were characterized by substantial brain inflammation were analyzed in this study. Tissues were provided by the UK Multiple Sclerosis Tissue Bank at Imperial College in London after collection via a prospective donor program with fully informed consent. Based on the available clinical documentation, all MS patients were in the progressive phase of the disease, and no immunotherapy is reported in the 6 months before death. Control tissues for BZLF-1 immunohistochemistry included fixed-frozen brain sections from one control subject who died for cardiac failure (obtained from the UK MS Tissue Bank), paraffin sections of a brain with tuberculous meningo-encephalitis and of an EBV-negative brain B-cell lymphoma (kindly provided by Dr R. Hoftberger, Clinical Institute of Neurology, Wien), and paraffin sections of a tonsil from a subject with active infectious mononucleosis (kindly provided by Dr G. Niedobitek, Sana Klinikum Lichtenberg/Unfallkrankenhaus, Berlin).

Eight brain tissue blocks (4 cm^3^ each; 1 snap frozen, 7 fixed frozen) from 5 MS cases (MS92, MS121, MS154, MS180, MS342) were used for immunohistochemical studies. Lesion inflammatory activity was assessed as previously described [63]. Five snap-frozen brain tissue blocks from 4 MS cases (MS79, MS92, MS180, MS342) were used to study BZLF-1 gene expression using quantitative real-time RT-PCR. One snap-frozen control lymph node was obtained from Dr Egidio Stigliano, Policlinico A. Gemelli, Rome.

### Immunohistochemistry and immunofluorescence

Brain sections were stained using immunohistochemical and single or double indirect immunofluorescence techniques. Immunohistochemical detection of CD20, MHC class II antigen and myelin-oligodendrocyte glycoprotein (MOG), and immunofluorescence stainings for BFRF1, Ig-A,-G,-M, CD8 and perforin alone or in different combinations were performed as previously described [32]. For BZLF-1 immunohistochemistry, deparaffinised sections from infectious mononucleosis tonsil, cerebral B cell lymphoma and brain with tuberculous meningo-encephalitis and cryosections from PFA-fixed control brain were subjected to antigen retrieval procedure in citrate buffer in microwave for 3 cycles of 3 min each before quenching of endogenous peroxidase activity in PBS containing 0,1% H_2_O_2_. Sections were treated with 0.5% Triton X-100 in PBS for 10 minutes and incubated for 1 hour with normal serum 30%+0,25% Triton X-100 and then overnight at 4°C with mouse monoclonal antibody (mAb) specific for BZLF-1 protein (clone BZ-1, kindly provided by Dr J. Middeldorp, VUMC, Amsterdam) diluted 1∶50 in PBS containing 0,25% Triton X-100. Sections were then incubated with biotin-conjugated rabbit anti-mouse antibody (Jackson Immunoreaearch Laboratories, West Grove, PA) for 1 hour at room temperature (RT), ABC-peroxidase complex (Vector Laboratories, Burlingame, CA) for 45 min, and AEC (DakoCytomation, Glostrup, Denmark) or diaminobenzidine (Sigma, St Louis, MO) to reveal the immune reaction.

The EBV-producing B95-8 cells (marmoset B-cell line transformed with EBV) [64] were used as positive control for BZLF-1 immunofluorescence staining. Paraformaldehyde-fixed frozen brain sections from MS cases and one control case were air-dried and post-fixed in 4% PFA for 5 minutes at RT or in iced acetone for 10 minutes at 4°C. Sections were subjected to antigen unmasking, permeabilization steps and block of unspecific binding sites as described above and then incubated for 36 h at 4°C with BZ-1 mAb (diluted 1∶50 in PBS +0,1% Triton X-100). Antibody binding was visualized using tetramethyl rhodamine isothiocyanate (TRITC)-conjugated goat anti-mouse antibody (Jackson Laboratories) containing 5% normal goat serum for 50 minutes at RT. After washing, sections were sealed in DAPI-containing medium or incubated further with FITC-conjugated rabbit anti-human Ig-A-G-M (1∶400; Dako Cytomation) for 1 hour at RT. For double immunofluorescence for BZLF-1 and CD8, sections were stained with BZ-1 mAb and anti-human CD8 rabbit polyclonal antibody (1∶50; Pierce, Thermo Fisher Scientific Inc. Rockford, IL) followed by a mixture of Alexa Fluor 488-conjugated goat anti-mouse and TRITC-conjugated goat anti-rabbit secondary antibodies. Double and triple immunostainings for CD8/granzyme B and CD8/granzyme B/BFRF1 were performed by incubating PFA-fixed cryosections with a mixture of anti-CD8 rabbit polyclonal antibody and anti-granzyme B mAb (Dako), or anti-CD8 mAb, anti-BFRF1 rabbit polyclonal (1∶800) and anti-granzyme B goat polyclonal (1∶50, Santa Cruz) antibodies overnight at 4°C, and then with a mixture of donkey FITC anti-mouse (Invitrogen, Eugene, OR), TRITC anti-rabbit and AMCA anti-goat (Jackson Immunoresearch Lab) secondary antibodies. Sections were sealed in ProLong Gold antifade reagent with 4′,6′-diamidino-2-phenylindole (DAPI) (Invitrogen) or in Vectashield (Vector Laboratories). Images were analysed and acquired with a digital epifluorescence microscope (Leica Microsystem, Wetzlar, Germany). Negative control stainings were performed using Ig isotype controls and/or pre-immune sera.

### Laser capture microdissection

Snap-frozen brain tissue blocks from 4 MS cases (MS79, MS92, MS180, MS342) and control lymph node were used for laser capture microdissection and subsequent RNA analysis. For each tissue block, the integrity and quality of total RNA extracted with the SV Total RNA Isolation System (Promega, Madison, WI) were checked on ethidium bromide containing 1% agarose gels in Tris-borate/EDTA buffer. Ten to 20 serial brain sections for each MS case and from control lymph-node were mounted on membrane slides for laser capture microdissection (MMI AG, Glattbrugg, Switzerland) and processed as described previously [33]. Sections before and after these series were stained for CD20 and Ig to identify B cell- and plasma cell-containing regions in the inflamed meninges and white matter lesions. Using a laser microdissector SL Cut (MMI AG) equipped with a Nikon Eclipse TE2000-S microscope, we isolated areas containing meningeal infiltrates and B-cell follicles, lesioned grey matter, B cell-enriched perivascular cuffs in white matter lesions, lesioned white matter surrounding inflamed blood vessels, and normal-appearing white matter. The same brain areas were cut in 3 to 10 serial sections and pooled in a single cap. B cell follicles were isolated from the lymph-node. The isolated tissue fragments were collected in 50 µL of lysis buffer (PicoPure RNA isolation kit, Arcturus Engineering), incubated at 42C° for 30 minutes and centrifuged at 800× g for 2 minutes. Lysates were stored at −80 C° until use.

### RNA isolation and quantitative real time RT-PCR

DNase-treated total RNA was extracted from 20-µm-thick brain sections or microdissected areas from 4 MS brains and 1 control lymph node, as previously described [32]. RNA samples were reverse-transcribed with oligo (dT) and random hexamers using the Murine Leukemia Virus Reverse Transcriptase (Invitrogen Life Technologies, Carlsbad, CA). PreAMP Master Mix Kit (Applied Biosystems, Foster City, CA) was used to enrich for both cellular and viral gene transcripts. The cDNAs obtained from whole brain sections and microdissected brain and lymph node samples were preamplified according to the manufacturers' instructions using 90 nmol/L of each primer in a mix containing the same forward and reverse primers for GAPDH, CD19 and BZLF-1 used for real time RT-PCR. Quantitative PCR assays were performed in triplicate, as previously described [33]. cDNA from EBV-positive P3HR-1 cells and human primary B cells were included in each run as positive controls for BZLF-1 and CD19 gene expression, respectively. Sample values were normalized by calculating the relative quantity of each mRNA to that of GAPDH using the formula 2^−ΔCt^, where ΔCt represents the difference in cycle threshold (Ct) between target mRNA and GAPDH mRNA. The following primer pairs were used in this study: GAPDH_for ACAGTCCATGCCATCACTGCC; GAPDH_rev GCCTGCTTCACCACCTTCTTG [33]; BZLF-1_for GTTGTGGTTTCCGTGTGC; BZLF-1_rev AACAGCTAGCAGACATTGGTG [65]; CD19_for AGAACCAGTACGGGAACGTG; CD19_rev CTGCTCGGGTTTCCATAAGA [33].

### Statistical analysis

Differences between categorical variables were evaluated by Pearson's chi-squared test while differences between continuous variables were analysed by unpaired t-test with 95% confidence intervals.

## Supporting Information

Figure S1 **Flow cytometric analysis of EBV-specific CD8+ T cells.** Examples of flow cytometric profiles demonstrating the gating strategy to identify live CD8+ T cells specific for one of the EBV peptides (BZLF1) tested. The threshold for pentamer positivity was set at >0.02% of CD3+ cells. The number in the right panel indicates the percentage of pentamer+ cells within the CD3+ T cell population.
(TIF)

Figure S2 **Prevalence of EBV- and CMV- specific CD8+ T cell responses in HLA-A2+ HD and MS patients.** (A) HLA-A*0201 pentamer+ CD8+ T cells specific for latent (LMP-2A) and lytic (BMLF-1) antigens (left panel) and for CMV antigen (pp65) (right panel) were investigated in HLA-A2+ HD (n = 34) and MS patients (n = 45). No differences were found in the percentages of HD and MS patients with EBV- and CMV-specific CD8+ T cells over the threshold for pentamer positivity. (B) When patients were stratified according to disease activity (active MS = a-MS, n = 18; inactive MS = i-MS, n = 27), the proportion of inactive MS patients with a detectable EBV-specific CD8+ T cell response tended to be lower than that of HD and active MS patients (left panel). In contrast, no differences in the prevalence of CMV-specific CD8+ T cell responses were found between HD, inactive MS and active MS patients (right panel). The percentages of individuals with detectable EBV or CMV pentamer+ CD8+ T cells (grey columns) among the total donors tested (white columns) are shown; p values were calculated with Pearson's chi-squared test.
(TIF)

Figure S3 **Lack of differences in the magnitude of EBV-and CMV-specific CD8+ T cell responses between HLA-A2+ healthy donors and MS patients.** (A) The frequencies of CD8+ T cells specific for EBV latent (LMP-2A) and lytic (BMLF-1) antigens and for CMV antigen (pp65) were analyzed in HLA-A2+ HD (n = 17) and MS patients (n = 16) by staining with the corresponding peptide/HLA-A*0201 pentamers. The percentages of pentamer+ cells were calculated after gating on total CD3+CD8+ T cells. No differences were found in the frequencies of EBV- and CMV-specific CD8+ T cells between HD and total MS patients. Bars represent the median ± the minimum and maximum value. (B) Similar frequencies of CMV-specific CD8+ T cells were found in HD (n = 9), active MS (a-MS n = 6) and inactive MS (i-MS n = 7) patients. Data in logarithmic scale and mean values ± SD are shown; p values are calculated with unpaired t-test with 95% confidence intervals. (C) Examples of flow cytometric profiles for pentamer+ CD8+ T cells specific for CMV antigen in HD, active and inactive MS patients. The numbers represent the percentages of pentamer+ cells within the CD3+ CD8+ T-cell population.
(TIF)

Figure S4 **Lack of correlation between frequency of EBV-specific CD8+ T cells and MS disease duration.** Disease duration (x-axis) was correlated with the frequencies of CD8+ T cells specific for the EBV latent and lytic antigens tested (y-axis) in inactive MS (n = 13) (left panel) and active MS (n = 13) (right panel) patients. Each symbol represents the individual response to a different EBV antigen. No statistically significant correlation was found between the frequency of EBV-specific CD8+ T cells and disease duration in both patient groups (Spearman's coefficient r).
(TIF)

Figure S5 **Immunostaining for BZLF-1 protein in control cells and tissues.** A) EBV-producing B95-8 cells [marmoset B-cell line transformed with EBV (Miller G. and A. Lipman. Proc.Natl.Acad.Sci. USA 70: 190–194, 1973)] were induced for 48 h with 12-O-tetradecanoylphorbol-13-acetate (20 ng/ml) and sodium butyrate (3 mM) to activate viral replication, and used as positive control for BZLF-1 immunofluorescence staining. Many cells are positive for BZLF-1 (localized in the nucleus, red staining); cell nuclei are visualized with DAPI stain (blue). The inset shows a BZLF-1+ nucleus at high magnification. B) Immunostaining for BZLF-1 in a tonsil from a patient with infectious mononucleosis (nuclear brown signals); high magnification of a BZLF-1+ cell is shown in the inset. Absence of BZLF-1 immunostaining in brain sections from a control case, died for cardiac failure (C), from a patient with tuberculous meningoencephalitis (D) and in an EBV-negative cerebral B-cell lymphoma (E). Bars: 200 µm in C-E; 50 µm in B; 20 µm in A and inset in B; 10 µm in the inset in A.
(TIF)

## References

[1] Compston A, Coles A (2008) Multiple sclerosis. Lancet 372: 1502–1517. 18970977 10.1016/S0140-6736(08)61620-7

[2] Nylander A, Hafler DA (2012) Multiple sclerosis. J Clin Invest 122: 1180–1188. 22466660 10.1172/JCI58649 PMC3314452

[3] Lünemann JD, Münz C (2009) EBV in MS: guilty by association? Trends Immunol 30: 243–248. 19428300 10.1016/j.it.2009.03.007

[4] Ascherio A, Munger KL (2010) Epstein-Barr virus infection and multiple sclerosis: a review. J Neuroimmune Pharmacol 5: 271–277. 20369303 10.1007/s11481-010-9201-3

[5] Pender MP (2011) The essential role of Epstein-Barr virus in the pathogenesis of multiple sclerosis. Neuroscientist 17: 351–367. 21075971 10.1177/1073858410381531 PMC3764840

[6] Thorley-Lawson DA (2001) Epstein-Barr virus: exploiting the immune system. Nat Rev Immunol 1: 75–82. 11905817 10.1038/35095584

[7] James JA, Robertson JM (2012) Lupus and epstein-barr. Curr Opin Rheumatol 24: 383–388. 22504579 10.1097/BOR.0b013e3283535801 PMC3562348

[8] Posnett DN (2008) Herpesviruses and autoimmunity. Curr Opin Investig Drugs 9: 505–514. 18465661

[9] Sumaya CV, Myers LW, Ellison GW, Ench Y (1985) Increased prevalence and titer of Epstein-Barr virus antibodies in patients with multiple sclerosis. Ann Neurol 17: 371–377. 2988410 10.1002/ana.410170412

[10] Sundström P, Juto P, Wadell G, Hallmans G, Svenningsson A, et al (2004) An altered immune response to Epstein-Barr virus in multiple sclerosis: a prospective study. Neurology 62: 2277–2282. 15210894 10.1212/01.wnl.0000130496.51156.d7

[11] Levin LI, Munger KL, Rubertone MV, et al (2005) Temporal relationship between elevation of Epstein-Barr virus antibody titers and initial onset of neurological symptoms in multiple sclerosis. JAMA 293: 2496–500. 15914750 10.1001/jama.293.20.2496

[12] Banwell B, Krupp L, Kennedy J, Tellier R, Tenembaum S, et al (2007) Clinical features and viral serologies in children with multiple sclerosis: a multinational observational study. Lancet Neurol 6: 773–781. 17689148 10.1016/S1474-4422(07)70196-5

[13] Lünemann JD, Huppke P, Roberts S, Brück W, Gärtner J, Münz C (2008) Broadened and elevated humoral immune response to EBNA1 in pediatric multiple sclerosis. Neurology 71: 1033–1035. 18809840 10.1212/01.wnl.0000326576.91097.87 PMC2676958

[14] Mechelli R, Anderson J, Vittori D, Coarelli G, Annibali V, et al (2011) Epstein-Barr virus nuclear antigen-1 B-cell epitopes in multiple sclerosis twins. Mult Scler 17: 1290–1294. 21757535 10.1177/1352458511410515

[15] Ascherio A, Munger KL, Lennette ET, Spiegelman D, Hernán MA, et al (2001) Epstein-Barr virus antibodies and risk of multiple sclerosis: a prospective study. JAMA 286: 3083–3088. 11754673 10.1001/jama.286.24.3083

[16] Lünemann JD, Tintoré M, Messmer B, Strowig T, Rovira A, et al (2010) Elevated Epstein-Barr virus-encoded nuclear antigen-1 immune responses predict conversion to multiple sclerosis. Ann Neurol 67: 159–169. 20225269 10.1002/ana.21886 PMC2848293

[17] Handel AE, Williamson AJ, Disanto G, Handunnetthi L, Giovannoni G, et al (2010) an updated meta-analysis of risk of multiple sclerosis following infectious mononucleosis. PLoS One 5 (9) e12496. 20824132 10.1371/journal.pone.0012496 PMC2931696

[18] Lünemann JD, Edwards N, Muraro PA, Hayashi S, Cohen JI, et al (2006) Increased frequency and broadened specificity of latent EBV nuclear antigen-1-specific T cells in multiple sclerosis. Brain 129: 1493–506. 16569670 10.1093/brain/awl067

[19] Höllsberg P, Hansen HJ, Haahr S (2003) Altered CD8+ T cell responses to selected Epstein-Barr virus immunodominant epitopes in patients with multiple sclerosis. Clin Exp Immunol 132: 137–143. 12653848 10.1046/j.1365-2249.2003.02114.x PMC1808679

[20] Cepok S, Zhou D, Srivastava R, Nessler S, Stei S, et al (2005) Identification of Epstein-Barr virus proteins as putative targets of the immune response in multiple sclerosis. J Clin Invest 115: 1352–1360. 15841210 10.1172/JCI23661 PMC1077174

[21] Jilek S, Schluep M, Meylan P, Vingerhoets F, Guignard L, et al (2008) Strong EBV-specific CD8+ T-cell response in patients with early multiple sclerosis. Brain 131: 1712–1721. 18550621 10.1093/brain/awn108

[22] Pender MP, Csurhes PA, Lenarczyk A, Pfluger CM, Burrows SR (2009) Decreased T cell reactivity to Epstein-Barr virus infected lymphoblastoid cell lines in multiple sclerosis. J Neurol Neurosurg Psychiatry 80: 498–505. 19015225 10.1136/jnnp.2008.161018 PMC2663364

[23] Jilek S, Schluep M, Harari A, Canales M, Lysandropoulos A, et al (2012) HLA-B7-restricted EBV-specific CD8+ T cells are dysregulated in multiple sclerosis. J Immunol 188: 4671–4680. 22461701 10.4049/jimmunol.1103100

[24] Pender MP (2003) Infection of autoreactive B lymphocytes with EBV, causing chronic autoimmune diseases. Trends Immunol 24: 584–588. 14596882 10.1016/j.it.2003.09.005

[25] Lünemann JD, Jelcić I, Roberts S, Lutterotti A, Tackenberg B, et al (2008) EBNA1-specific T cells from patients with multiple sclerosis cross react with myelin antigens and co-produce IFN-gamma and IL-2. J Exp Med 205: 1763–1773. 18663124 10.1084/jem.20072397 PMC2525578

[26] Lang HL, Jacobsen H, Ikemizu S, Andersson C, Harlos K, et al (2002) A functional and structural basis for TCR cross-reactivity in multiple sclerosis. Nat Immunol 3: 940–943. 12244309 10.1038/ni835

[27] Lassmann H, Niedobitek G, Aloisi F, Middeldorp JM (2011) NeuroproMiSe EBV Working Group (2011) Epstein-Barr virus in the multiple sclerosis brain: a controversial issue - report on a focused workshop held in the Centre for Brain Research of the Medical University of Vienna, Austria. Brain 134: 2772–2786. 21846731 10.1093/brain/awr197 PMC3170536

[28] Peferoen LA, Lamers F, Lodder LN, Gerritsen WH, Huitinga I, et al (2010) Epstein Barr virus is not a characteristic feature in the central nervous system in established multiple sclerosis. Brain 133: 1–4. 19917644 10.1093/brain/awp296

[29] Willis SN, Stadelmann C, Rodig SJ, et al (2009) Epstein-Barr virus infection is not a characteristic feature of multiple sclerosis brain. Brain 132: 3318–3328. 19638446 10.1093/brain/awp200 PMC2792367

[30] Torkildsen O, Stansberg C, Angelskar SM, et al (2010) Upregulation of immunoglobulin-related genes in cortical sections from multiple sclerosis patients. Brain Pathol 20: 720–729. 19919606 10.1111/j.1750-3639.2009.00343.x PMC8094770

[31] Sargsyan SA, Shearer AJ, Ritchie AM, et al (2010) Absence of Epstein-Barr virus in the brain and CSF of patients with multiple sclerosis. Neurology 74: 1127–1135. 20220124 10.1212/WNL.0b013e3181d865a1 PMC2865779

[32] Serafini B, Rosicarelli B, Franciotta D, Magliozzi R, Reynolds R, et al (2007) Dysregulated Epstein Barr virus infection in the multiple sclerosis brain. J Exp Med 204: 2899–2912. 17984305 10.1084/jem.20071030 PMC2118531

[33] Serafini B, Severa M, Columba-Cabezas S, Rosicarelli B, Veroni C, et al (2010) Epstein-Barr virus latent infection and BAFF expression in B cells in the multiple sclerosis brain: implications for viral persistence and intrathecal B-cell activation. J Neuropathol Exp Neurol 69: 677–693. 20535037 10.1097/NEN.0b013e3181e332ec

[34] Serafini B, Muzio L, Rosicarelli B, Aloisi F (2013) Radioactive in situ hybridization for EBER supports presence of Epstein-Barr virus in the multiple sclerosis brain. Brain doi: 10.1093/brain/aws315. 10.1093/brain/aws315 23355688

[35] Tzartos JS, Khan G, Vossenkamper A, Cruz-Sadaba M, Lonardi S, et al (2012) Association of innate immune activation with latent Epstein-Barr virus in active MS lesions. Neurology 78: 15–23. 22156987 10.1212/WNL.0b013e31823ed057

[36] Farrell RA, Antony D, Wall GR, Clark DA, Fisniku L, et al (2009) Humoral immune response to EBV in multiple sclerosis is associated with disease activity on MRI. Neurology 73: 32–38. 19458321 10.1212/WNL.0b013e3181aa29fe PMC2848585

[37] Buljevac D, van Doornum GJ, Flach HZ, Groen J, Osterhaus AD, et al (2005) Epstein-Barr virus and disease activity in multiple sclerosis. J Neurol Neurosurg Psychiatry 76: 1377–1381. 16170080 10.1136/jnnp.2004.048504 PMC1739347

[38] Murray RJ, Kurilla MG, Brooks JM, Thomas WA, Rowe M, et al (1992) Identification of target antigens for the human cytotoxic T cell response to Epstein-Barr virus (EBV): implications for the immune control of EBV-positive malignancies. J Exp Med 176: 157–168. 1319456 10.1084/jem.176.1.157 PMC2119296

[39] Steven NM, Annels NE, Kumar A, Leese AM, Kurilla MG, et al (1997) Immediate early and early lytic cycle proteins are frequent targets of the Epstein-Barr virus-induced cytotoxic T cell response. J Exp Med 185: 1605–1617. 9151898 10.1084/jem.185.9.1605 PMC2196300

[40] Palendira U, Chinn R, Raza W, Piper K, Pratt G, et al (2008) Selective accumulation of virus-specific CD8+ T cells with unique homing phenotype within the human bone marrow. Blood 112: 3293–3302. 18635810 10.1182/blood-2008-02-138040

[41] Kieseier BC (2011) The mechanism of action of interferon-β in relapsing multiple sclerosis. CNS Drugs 25: 491–502. 21649449 10.2165/11591110-000000000-00000

[42] Rudick R, Polman C, Clifford D, Miller D, Steinman L (2012) Natalizumab: Bench to Bedside and Beyond. Arch Neurol 5: 1–11. 10.1001/jamaneurol.2013.598 23128399

[43] Laichalk LL, Thorley-Lawson DA (2005) Terminal differentiation into plasma cells initiates the replicative cycle of Epstein-Barr virus in vivo. J Virol 79: 1296–1307. 15613356 10.1128/JVI.79.2.1296-1307.2005 PMC538585

[44] Farina A, Santarelli R, Gonnella R, Bei R, Muraro R, et al (2000) The BFRF1 gene of Epstein-Barr virus encodes a novel protein. J Virol 74: 3235–3244. 10708440 10.1128/jvi.74.7.3235-3244.2000 PMC111824

[45] Hislop AD, Taylor GS, Sauce D, Rickinson AB (2007) Cellular responses to viral infection in humans: lessons from Epstein-Barr virus. Annu Rev Immunol 25: 587–617. 17378764 10.1146/annurev.immunol.25.022106.141553

[46] Wherry EJ, Ha SJ, Kaech SM, Haining WN, Sarkar S, et al (2007) Molecular signature of CD8+ T cell exhaustion during chronic viral infection. Immunity 27: 670–684. 17950003 10.1016/j.immuni.2007.09.006

[47] Sauce D, Larsen M, Abbott RJ, Hislop AD, Leese AM, et al (2009) Upregulation of interleukin 7 receptor alpha and programmed death 1 marks an epitope-specific CD8+ T-cell response that disappears following primary Epstein-Barr virus infection. J Virol 83: 9068–9078. 19605492 10.1128/JVI.00141-09 PMC2738242

[48] Gross AJ, Hochberg D, Rand WM, Thorley-Lawson DA (2005) EBV and systemic lupus erythematosus: a new perspective. J Immunol 174: 6599–6607. 15905498 10.4049/jimmunol.174.11.6599

[49] Larsen M, Sauce D, Deback C, Arnaud L, Mathian A, et al (2011) Exhausted cytotoxic control of Epstein-Barr virus in human lupus. PLoS Pathog 7: e1002328 Epub 2011 Oct 20. 22028659 10.1371/journal.ppat.1002328 PMC3197610

[50] Comabella M, Kakalacheva K, Rio J, Münz C, Montalban X, et al (2012) EBV-specific immune responses in patients with multiple sclerosis responding to IFNβ therapy. Mult Scler J 18: 605–609. 10.1177/1352458511426816 22020417

[51] Frisullo G, Iorio R, Plantone D, Marti A, Nociti V, et al (2011) CD4+T-bet+, CD4+pSTAT3+ and CD8+T-bet+ T cells accumulate in peripheral blood during NZB treatment. Mult Scler 17: 556–566. 21177324 10.1177/1352458510392263

[52] Means RE, Lang SM, Jung JU. (2007) Human gammaherpesvirus immune evasion strategies. In: Arvin A, Campadelli-Fiume G, Mocarski E, Moore PS, Roizman B, Whitley R, Yamanishi K, editors. Human Herpesviruses: Biology, Therapy, and Immunoprophylaxis. Cambridge: Cambridge University Press. Chapter 31.

[53] Jaquiéry E, Jilek S, Schluep M, Meylan P, Lysandropoulos A, et al (2010) Intrathecal immune responses to EBV in early MS. Eur J Immunol 40: 878–887. 20017197 10.1002/eji.200939761

[54] Malmeström C, Lycke J, Haghighi S, Andersen O, Carlsson L, et al (2008) Relapses in multiple sclerosis are associated with increased CD8+ T-cell mediated cytotoxicity in CSF. J Neuroimmunol 196: 159–165. 18396337 10.1016/j.jneuroim.2008.03.001

[55] Babbe H, Roers A, Waisman A, Lassmann H, Goebels N, et al (2000) Clonal expansions of CD8(+) T cells dominate the T cell infiltrate in active multiple sclerosis lesions as shown by micromanipulation and single cell polymerase chain reaction. J Exp Med 192: 393–404. 10934227 10.1084/jem.192.3.393 PMC2193223

[56] Skulina C, Schmidt S, Dornmair K, Babbe H, Roers A, et al (2004) Multiple sclerosis: brain-infiltrating CD8+ T cells persist as clonal expansions in the cerebrospinal fluid and blood. Proc Natl Acad Sci U S A 101: 2428–2433. 14983026 10.1073/pnas.0308689100 PMC356967

[57] Foxman EF, Iwasaki A (2011) Genome-virome interactions: examining the role of common viral infections in complex disease. Nature Rev Microbiol 9: 254–264. 21407242 10.1038/nrmicro2541 PMC3678363

[58] Recher M, Lang KS, Navarini A, Hunziker L, Lang PA, et al (2007) Extralymphatic virus sanctuaries as a consequence of potent T-cell activation. Nat Med 13: 1316–1323. 17982463 10.1038/nm1670 PMC7096094

[59] Lycke J, Svennerholm B, Hjelmquist E, Frisén L, Badr G, et al (1996) Acyclovir treatment of relapsing-remitting multiple sclerosis. A randomized, placebo-controlled, double-blind study. J Neurol 243: 214–224. 8936350 10.1007/BF00868517

[60] Bech E, Lycke J, Gadeberg P, Hansen HJ, Malmeström C, et al (2002) A randomized, double-blind, placebo-controlled MRI study of anti-herpes virus therapy in MS. Neurology 58: 31–36. 11781402 10.1212/wnl.58.1.31

[61] Dreyfus DH (2011) Autoimmune disease: A role for new anti-viral therapies? Autoimmun Rev 11: 88–97. 21871974 10.1016/j.autrev.2011.08.005

[62] Polman CH, Reingold SC, Edan G, Filippi M, Hartung HP, et al (2005) Diagnostic criteria for multiple sclerosis: 2005 revisions to the “McDonald Criteria”. Ann Neurol 58: 840–846. 16283615 10.1002/ana.20703

[63] Magliozzi R, Howell O, Vora A, Serafini B, Nicholas R, et al (2007) Meningeal B-cell follicles in secondary progressive multiple sclerosis associate with early onset of disease and severe cortical pathology. Brain 130: 1089–1104. 17438020 10.1093/brain/awm038

[64] Miller G, Lipman MJ (1973) Release of infectious Epstein-Barr virus by transformed marmoset leukocytes. Natl Acad Sci U S A 70: 190–194. 10.1073/pnas.70.1.190 PMC433213 4346033

[65] Lindsey JW, Hatfield LM, Crawford MP, Patel S (2009) Quantitative PCR for Epstein-Barr virus DNA and RNA in multiple sclerosis. Mult Scler 15: 153–158. 18845656 10.1177/1352458508097920

