# Supplementary figures and images for: Increased CD8+ T Cell Response to Epstein-Barr Virus Lytic Antigens in the Active Phase of Multiple Sclerosis
Source: PLoS Pathog. 2013 Apr 11;9(4):e1003220. doi: 10.1371/journal.ppat.1003220 (PMC3623710; doi:10.1371/journal.ppat.1003220)

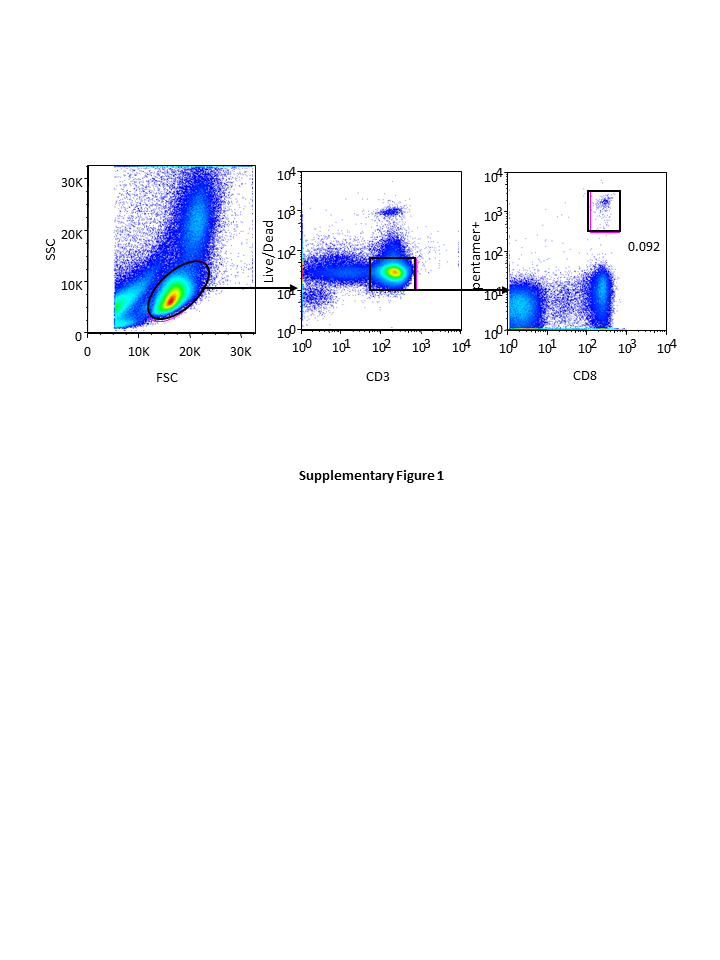

Supplement: Figure S1 — Flow cytometric analysis of EBV-specific CD8+ T cells. Examples of flow cytometric profiles demonstrating the gating strategy to identify live CD8+ T cells specific for one of the EBV peptides (BZLF1) tested. The threshold for pentamer positivity was set at >0.02% of CD3+ cells. The number in the right panel indicates the percentage of pentamer+ cells within the CD3+ T cell population. (TIF) [file ppat.1003220.s001.tif]

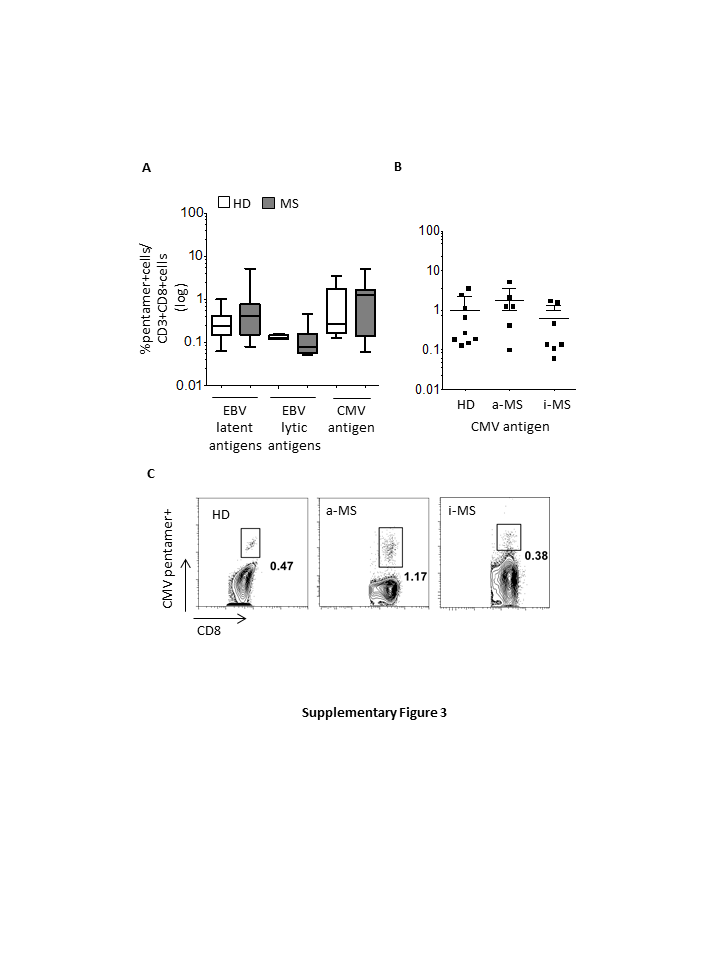

Supplement: Figure S3 — Lack of differences in the magnitude of EBV-and CMV-specific CD8+ T cell responses between HLA-A2+ healthy donors and MS patients. (A) The frequencies of CD8+ T cells specific for EBV latent (LMP-2A) and lytic (BMLF-1) antigens and for CMV antigen (pp65) were analyzed in HLA-A2+ HD (n = 17) and MS patients (n = 16) by staining with the corresponding peptide/HLA-A*0201 pentamers. The percentages of pentamer+ cells were calculated after gating on total CD3+CD8+ T cells. No differences were found in the frequencies of EBV- and CMV-specific CD8+ T cells between HD and total MS patients. Bars represent the median ± the minimum and maximum value. (B) Similar frequencies of CMV-specific CD8+ T cells were found in HD (n = 9), active MS (a-MS n = 6) and inactive MS (i-MS n = 7) patients. Data in logarithmic scale and mean values ± SD are shown; p values are calculated with unpaired t-test with 95% confidence intervals. (C) Examples of flow cytometric profiles for pentamer+ CD8+ T cells specific for CMV antigen in HD, active and inactive MS patients. The numbers represent the percentages of pentamer+ cells within the CD3+ CD8+ T-cell population. (TIF) [file ppat.1003220.s003.tif]

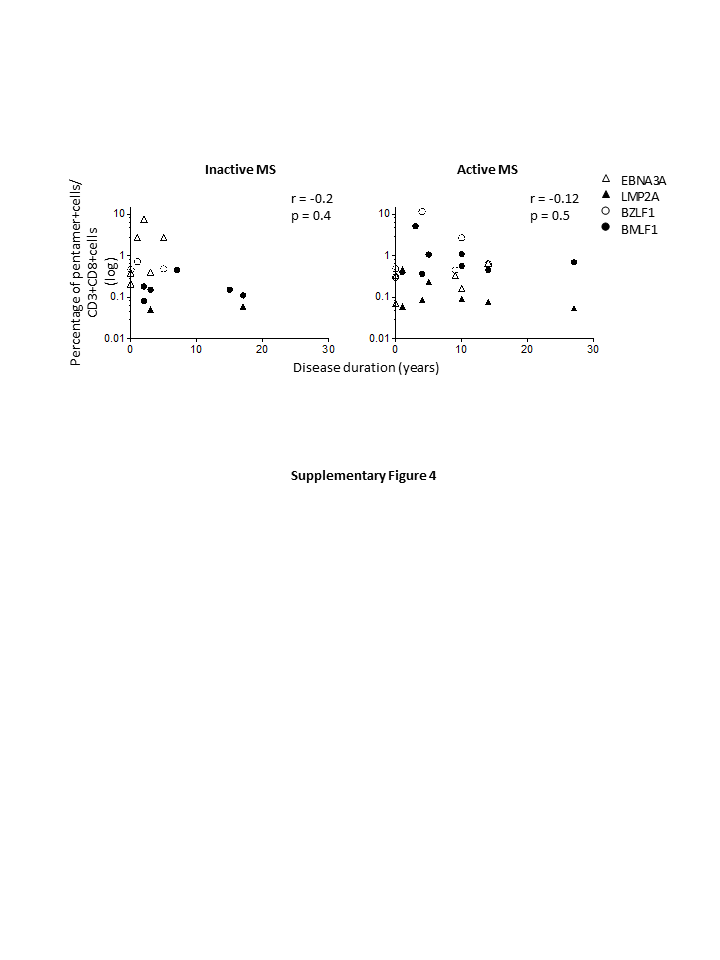

Supplement: Figure S4 — Lack of correlation between frequency of EBV-specific CD8+ T cells and MS disease duration. Disease duration (x-axis) was correlated with the frequencies of CD8+ T cells specific for the EBV latent and lytic antigens tested (y-axis) in inactive MS (n = 13) (left panel) and active MS (n = 13) (right panel) patients. Each symbol represents the individual response to a different EBV antigen. No statistically significant correlation was found between the frequency of EBV-specific CD8+ T cells and disease duration in both patient groups (Spearman's coefficient r). (TIF) [file ppat.1003220.s004.tif]

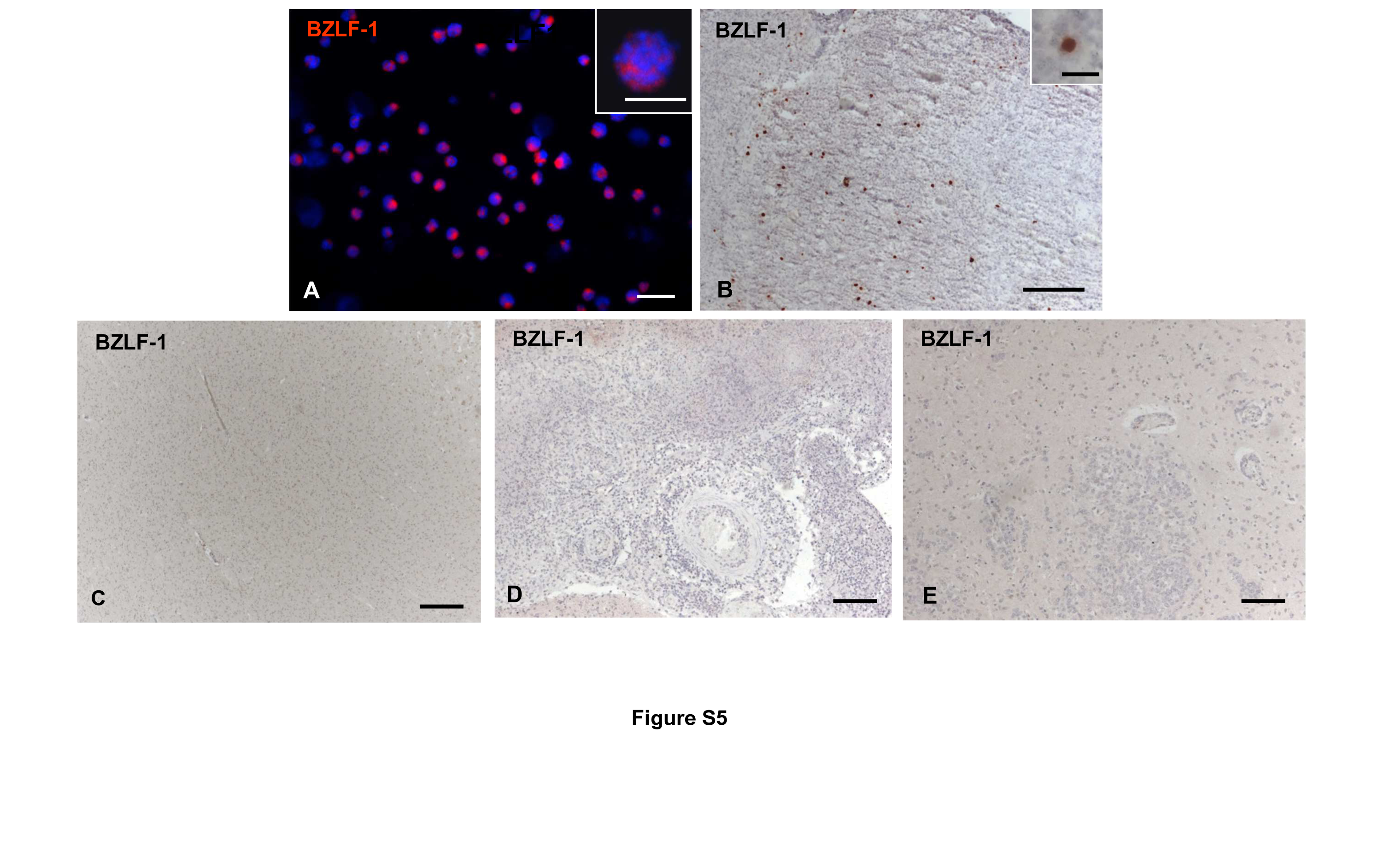

Supplement: Figure S5 — Immunostaining for BZLF-1 protein in control cells and tissues. A) EBV-producing B95-8 cells [marmoset B-cell line transformed with EBV (Miller G. and A. Lipman. Proc.Natl.Acad.Sci. USA 70: 190–194, 1973)] were induced for 48 h with 12-O-tetradecanoylphorbol-13-acetate (20 ng/ml) and sodium butyrate (3 mM) to activate viral replication, and used as positive control for BZLF-1 immunofluorescence staining. Many cells are positive for BZLF-1 (localized in the nucleus, red staining); cell nuclei are visualized with DAPI stain (blue). The inset shows a BZLF-1+ nucleus at high magnification. B) Immunostaining for BZLF-1 in a tonsil from a patient with infectious mononucleosis (nuclear brown signals); high magnification of a BZLF-1+ cell is shown in the inset. Absence of BZLF-1 immunostaining in brain sections from a control case, died for cardiac failure (C), from a patient with tuberculous meningoencephalitis (D) and in an EBV-negative cerebral B-cell lymphoma (E). Bars: 200 µm in C-E; 50 µm in B; 20 µm in A and inset in B; 10 µm in the inset in A. (TIF) [file ppat.1003220.s005.tif]
